# Candidate‐species delimitation in *Desmognathus* salamanders reveals gene flow across lineage boundaries, confounding phylogenetic estimation and clarifying hybrid zones

**DOI:** 10.1002/ece3.8574

**Published:** 2022-02-16

**Authors:** Robert Alexander Pyron, Kyle A. O’Connell, Emily Moriarty Lemmon, Alan R. Lemmon, David A. Beamer

**Affiliations:** ^1^ Department of Biological Sciences The George Washington University Washington District of Columbia USA; ^2^ Division of Amphibians and Reptiles Department of Vertebrate Zoology National Museum of Natural History Smithsonian Institution Washington District of Columbia USA; ^3^ Global Genome Initiative National Museum of Natural History Smithsonian Institution Washington District of Columbia USA; ^4^ Biomedical Data Science Lab Deloitte Consulting LLP Arlington Virginia USA; ^5^ Department of Biological Science Florida State University Tallahassee Florida USA; ^6^ Department of Scientific Computing Florida State University Tallahassee Florida USA; ^7^ Department of Natural Sciences Nash Community College Rocky Mount North Carolina USA

**Keywords:** admixture, *Desmognathus*, hybrid zones, phylogenomics, species delimitation, systematics

## Abstract

Dusky Salamanders (genus *Desmognathus*) currently comprise only 22 described, extant species. However, recent mitochondrial and nuclear estimates indicate the presence of up to 49 candidate species based on ecogeographic sampling. Previous studies also suggest a complex history of hybridization between these lineages. Studies in other groups suggest that disregarding admixture may affect both phylogenetic inference and clustering‐based species delimitation. With a dataset comprising 233 Anchored Hybrid Enrichment (AHE) loci sequenced for 896 *Desmognathus* specimens from all 49 candidate species, we test three hypotheses regarding (i) species‐level diversity, (ii) hybridization and admixture, and (iii) misleading phylogenetic inference. Using phylogenetic and population‐clustering analyses considering gene flow, we find support for at least 47 candidate species in the phylogenomic dataset, some of which are newly characterized here while others represent combinations of previously named lineages that are collapsed in the current dataset. Within these, we observe significant phylogeographic structure, with up to 64 total geographic genetic lineages, many of which hybridize either narrowly at contact zones or extensively across ecological gradients. We find strong support for both recent admixture between terminal lineages and ancient hybridization across internal branches. This signal appears to distort concatenated phylogenetic inference, wherein more heavily admixed terminal specimens occupy apparently artifactual early‐diverging topological positions, occasionally to the extent of forming false clades of intermediate hybrids. Additional geographic and genetic sampling and more robust computational approaches will be needed to clarify taxonomy, and to reconstruct a network topology to display evolutionary relationships in a manner that is consistent with their complex history of reticulation.

## Graphical abstract

The Dusky Salamanders (genus Desmognathus) contain only 22 described species, but previous phylogenetic research suggests up to 49 candidate species. With a genome‐scale dataset, we find support for at least 47 species‐level lineages with a complex history of hybridization and admixture. These reticulations continue to confound attempts to untangle historical evolutionary relationships.

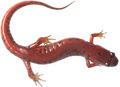

## 1 INTRODUCTION

Gene flow across the boundaries of even distantly related species is now recognized as a common occurrence in many groups at both deep and recent timescales (Harrison & Larson, 2014; Larson et al., 2014; Nosil, 2008; Schield et al., 2019). These processes have numerous downstream effects, confounding our ability to infer bifurcating phylogenies (Leaché et al., 2014) and revealing that an evolutionary network is, therefore, a more accurate topology for many groups (Solís‐Lemus et al., 2016). Reticulations, rather than bifurcations, are consequently a common feature of the evolutionary relationships of many taxa (Blair & Ané, 2020). Similarly, genetic quantification of species boundaries now increasingly recognizes the likelihood of admixture between “completed” species and the possible existence of hybrid populations with distinct patterns of genomic ancestry (Chan et al., 2017).

However, several related challenges complicate accurate inference of these evolutionary processes at scale. First, species boundaries must be established to determine when and where introgression has occurred (Harrison & Larson, 2014). Species limits are best represented as continuums of divergence rather than discrete boundaries; instances of hybridization may, therefore, represent fuzzy empirical outcomes in many cases (Chan et al., 2022). Second, the signal for both recent and ancient gene flow may be unequally distributed within the genome and among taxa (Weisrock & Larson, 2006). In the most extreme cases, evidence may be erased from the nuclear genome by selection or drift, potentially leaving only captured mitochondrial haplotypes as evidence (Toews & Brelsford, 2012). Third, existing methods are highly constrained in their ability to estimate even moderately complex networks (Pardi & Scornavacca, 2015). Most commonly used algorithms are limited to level‐1 networks (defined as those not sharing any edges between reticulations) and cannot estimate multiple hybridization events that intersect or share branches between them (Allman et al., 2019).

**FIGURE 1:**
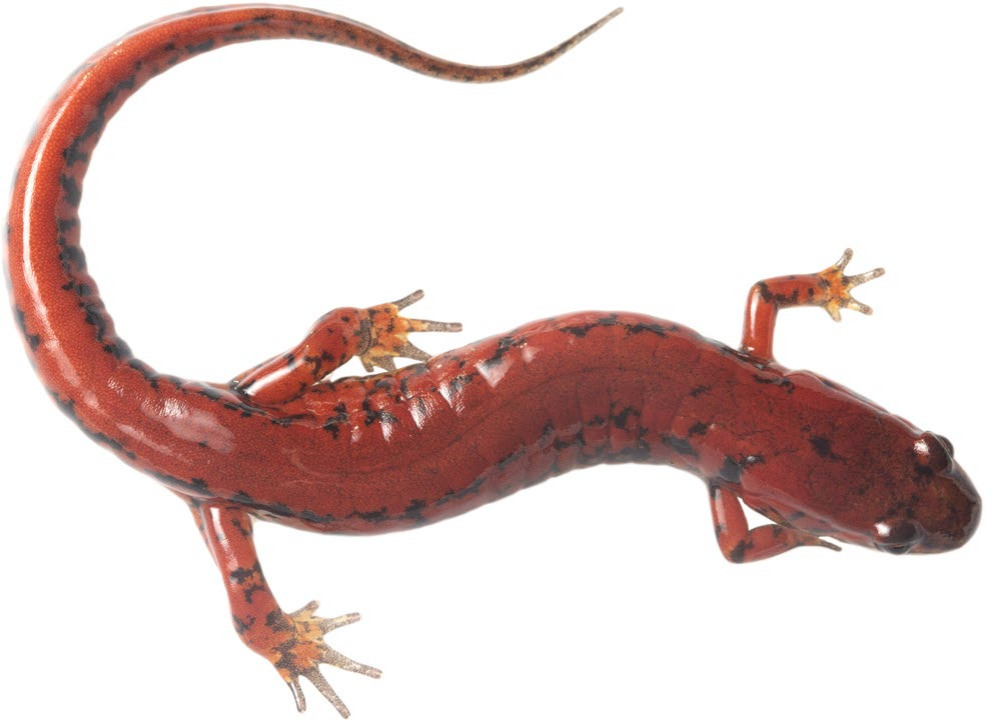
A specimen (RAP0890/NPS‐GRSM‐196373) from the *ocoee* “A” lineage of the Balsam clade (see below), exhibiting the unusual erythristic pigmentation seen in some populations of this candidate species on Cataloochee Balsam in the Great Smoky Mountains. The Balsam clade is characterized by possessing fossil mitochondria from an ancient instance of ‘ghost’ admixture (Lawson et al., 2018; Pyron et al., 2020; Zhang et al., 2019); such complex patterns are common in *Desmognathus*. Photo courtesy of T.W. Pierson

These conundrums are all evident in the plethodontid salamander genus *Desmognathus* (Figure 1). Of the 22 described species, several were morphologically cryptic and discovered only recently using molecular data (e.g., Camp et al., 2002). Many of the remaining morphospecies were discovered through further mitochondrial sequencing to represent polyphyletic assemblages (Kozak et al., 2005), with at least 45 mitochondrial lineages (Beamer & Lamb, 2020). Subsequent analyses (Pyron et al., 2020) supported the distinctiveness of at least 49 “mito‐nuclear candidate species” defined by ecogeographically monophyletic mitochondrial haplotypes and corroborated by genomic loci, revealing a complex history of reticulation involving both extant and ancestral lineages. However, these candidate species are based only on geography and phylogenetic or limited network analyses, and most have not yet been subjected to explicit delimitation analyses with population‐level sampling. Consequently, spatial boundaries and degrees of genealogical exclusivity are still undescribed for most lineages.

Additionally, the distribution and strength of the signal in the nuclear genome for the numerous known reticulation events has not been quantified. Some instances of nuclear admixture and mitochondrial capture are well known (Mead et al., 2001; Tilley et al., 2013). Others, such as a deep‐time reticulation involving the lineage ancestral to *aeneus* + *imitator,* were unexpected and not reflected in present‐day mitochondrial patterns (Pyron et al., 2020). Other clear instances of mitochondrial capture, such as *fuscus* E with haplotypes from *auriculatus* C (Beamer & Lamb, 2020), were not recovered by the preliminary network analyses. Therefore, a population‐level genomic assessment of species boundaries and admixture combined with known instances of mitochondrial capture is needed to quantify an accurate species delimitation model and an estimate of quantifiable hybridization events.

Finally, it is relatively unknown what effects both shallow and deep‐time reticulation events will have on estimation of both networks and phylogenies (Folk et al., 2016; Kutschera et al., 2014). While some early estimates suggested that species‐tree methods might be robust to modest amounts of gene flow (Leaché et al., 2014), many are now known to be inconsistent under these conditions (Solís‐Lemus et al., 2016), with particularly strong effects for rapid radiations (Jiao et al., 2020). Empirical descriptions of the effects gene flow may have on topological estimation and lineage‐based species delimitation are rare (Eckert & Carstens, 2008; McVay et al., 2017). Recent empirical work at the level of species and hybrid populations suggests a relatively straightforward effect: that admixed individuals often occupy artifactual positions on phylogenetic topologies (Chan et al., 2020). They create ladder‐like “grades” of intermediate topological position between the various parental lineages in relative proportion to their ratios of hybrid ancestry, which “attract” closely related non‐hybrids (Dolinay et al., 2021). As the history of *Desmognathus* is characterized by extensive cross‐lineage gene flow (Pyron et al., 2020), we can, thus, quantify how these processes affect estimates of phylogeny.

We use an expanded population‐level phylogenomic dataset to answer three primary questions in *Desmognathus* with broader relevance for understanding species delimitation across the phylogeography–phylogenetics continuum (Edwards et al., 2016). First, of the 49 mito‐nuclear candidate species, how many are supported by population‐genetic evidence from clustering methods that account for admixture between lineages (Frichot et al., 2014)? We anticipate that some candidate lineages may be collapsed, while other widespread lineages may contain significant phylogeographic structure that went undetected in previous analyses.

Second, which of these candidate species or phylogeographic lineages show evidence of hybridization across the nuclear and mitochondrial genomes, and what is the spatial extent of present‐day hybrid zones (Burbrink et al., 2021; Szymura & Barton, 1986)? The existence, location, and width of these geographic admixture zones may vary significantly among species pairs and loci (Barton, 1983; Dufresnes et al., 2020). Nonetheless, we anticipate geographic localization of heavily admixed individuals to coincide with the location of mito‐nuclear candidate species boundaries, aligned with physiographic features associated with climatic refugia and speciation in salamanders (Highton, 1995; Kozak & Wiens, 2006).

Third, how does the existence of recent admixture events between mito‐nuclear candidate species influence topological reconstructions? The impact of heavily admixed genomes on the terminal placement of individuals and their effect on the resolution of species‐level clades is now known to be significant in many cases (Chan et al., 2022; Dolinay et al., 2021). Given the prevalence of hybrid individuals in our sampled populations of *Desmognathus*, we hypothesize that at least some of the phylogenetic structure detected in mito‐nuclear candidate species by previous analyses may have been influenced by gene trees from these admixed terminal specimens, resulting in artifactual “clades” interpreted as meaningful units.

## 2 MATERIALS AND METHODS

### 2.1 Specimen sampling

**FIGURE 2:**
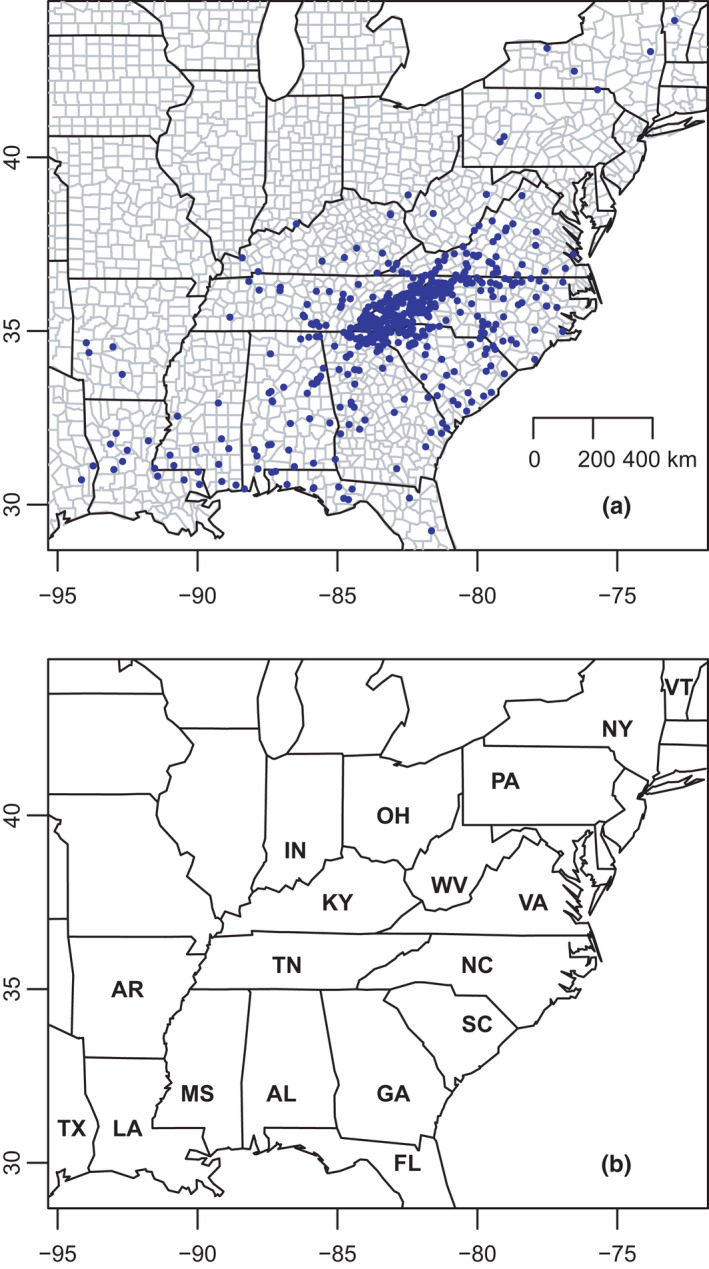
Map of the eastern United States, showing (a) the geographic location of 732 sampling sites in 18 states comprising 896 specimens from all 49 mito‐nuclear candidate species of *Desmognathus* and (b) the labels for those states: AL, Alabama, AR, Arkansas, FL, Florida, GA, Georgia, IN, Indiana, KY, Kentucky, LA, Louisiana, MS, Mississippi, NC, North Carolina, NY, New York, OH, Ohio, PA, Pennsylvania, SC, South Carolina, TN, Tennessee, TX, Texas, VA, Virginia, VT, Vermont, WV, West Virginia

Our previous studies (Beamer & Lamb, 2020; Pyron et al., 2020) included either a large number of samples (536) for a few mitochondrial genes (ND2, tRNAs, and COI), or a smaller number of individuals (161) for a larger number of loci (381 AHE genes). Based on our knowledge of the likely geographic extent of the 49 delimited mito‐nuclear candidate species and their potential hybrid zones informed by previous research (e.g., Tilley, 2016; Tilley et al., 2013), we expanded this sampling in the current dataset. We increased the representation of nearly all lineages, with 896 specimens nearly doubling the largest previous study, ranging from 1 to 92 individuals per clade (mean = 19) from 732 distinct sites in 18 states in the eastern United States (Figure 2). This includes nearly every known geographic population segment of *Desmognathus*, excepting a few marginal populations that are presumed extirpated. We also sequenced two formalin‐fixed, fluid‐preserved museum specimens (see Pyron et al., 2022) and only included these in a subset of analyses. Consequently, our primary sampling consisted of 894 specimens representing 49 mito‐nuclear candidate species. Based on geography or other preliminary analysis of mitochondrial or nuclear data, we assigned each of the 896 individuals to the 49 previously delimited groups, whereas we reassigned group membership of some individuals prior to the main clustering and admixture tests (see below). We performed part of the analyses on the GWU HPC *Pegasus* cluster (MacLachlan et al., 2020).

### 2.2 Anchored Hybrid Enrichment data

Data were generated using the Anchored Hybrid Enrichment (AHE) approach (Lemmon et al., 2012) as described in Hime et al. (2020) using the “*Desmognathus* version 2.0” probe set from Pyron et al. (2020). Allele phase was determined using the calling procedure described in Pyron et al. (2016). Sequencing and assembly proceeded in two batches, the first containing 810 samples and yielding 245 loci, the second containing 94 samples and yielding 316 loci. Homologous sets of loci between the two sets were determined by assembling their consensus sequences. Loci present in both assemblies were then merged via re‐alignment using mafft version 7.475 (Katoh & Standley, 2013) with the FFT‐NS‐i algorithm (maximum 1000 iterations). To ensure quality of base calls for downstream analysis prior to alignment, the data were trimmed for quality by removing alleles with >80% missing data or >5% ambiguities. Individuals with >50% missing data or only a single allele called per locus were retained for gene‐tree analyses but discarded for SNP‐based population‐genetic inferences. Eight individuals were removed due to failure or contamination, and the two fluid‐preserved specimens were excluded from the primary analyses and evaluated separately (see below). The merged set of orthologous loci contained 894 individuals with data from up to 233 loci ranging from 1025 to 5234 bp, totaling up to 563,656 bp, resulting in a matrix which was 86.4% complete.

Two of the individuals included in the primary sequence capture protocol described above were from formalin‐fixed, fluid‐preserved specimens collected by Richard D. Highton in 1971 (USNM 468094–5; *Desmognathus auriculatus*, FL: Marion) from a population (Silver Glen Springs, Ocala National Forest) which is now believed to be extirpated (see Dodd, 1998). These were extracted following O’Connell et al. (2021). Reads were obtained for some AHE loci, but assemblies were typically short with low coverage. Therefore, these individuals were removed from the primary alignments. For further analysis, we created a reduced secondary alignment with all 896 terminals, pruned to the 27,763 bp from 129 loci called for these two individuals, that was 94.4% complete overall. We applied a limited set of the gene‐tree and SNP‐based analyses described below to confirm the placement of these samples in corroboration of their previously estimated genetic identity as *auriculatus* A (Pyron et al., 2022).

### 2.3 Analytical strategy and computational constraints

Our ability to unravel the complexity of *Desmognathus* relationships fully is confounded by several factors. We know from previous analyses (Pyron et al., 2020) that there are multiple reticulations in the phylogeny of the group, both ancestral (i.e., involving internal branches) and recent (among terminal species). There are also several apparent instances of hybridization revealed by mito‐nuclear discordance (Beamer & Lamb, 2020) which have not yet been detected by network analyses. Collectively, known or suspected crosses have occurred between closely related mito‐nuclear candidate species (e.g., various lineages of *quadramaculatus* and *marmoratus* in the Pisgah clade), distantly related species groups (e.g., *ocoee* F/G/H and ‘gamma;’ *fuscus* C and *carolinensis*), and deep‐time reticulations (e.g., fossil mitochondria of *ocoee* A, B, C, & D; ancient hybridization between the stem lineages of the Pisgah and Nantahala clades). Consequently, we strongly suspect that many relationships in the group are characterized by non‐level‐1 networks, and therefore cannot be estimated by current methods (Solís‐Lemus & Ané, 2016) and may not be identifiable (Pardi & Scornavacca, 2015).

Similarly, population‐genetic methods designed to estimate or test explicit demographic models incorporating complex evolutionary dynamics are often heavily constrained in their ability to handle more than a few populations or terminal species (Excoffier et al., 2013; Gutenkunst et al., 2009; Hey, 2010; Jackson et al., 2017). Additionally, those methods often perform best with large numbers (i.e., thousands) of independent loci, whereas our sampling is limited to 233, despite our long total alignment. We also lack a reference genome to pinpoint significant patterns of chromosomal admixture and genomic differentiation (Gante et al., 2016; Li et al., 2019). Consequently, computational and data constraints prevent the ideal outcome of simultaneous inference of an adequately complex network and sufficiently parameterized species‐delimitation model. For these reasons, we apply a series of simple but robust procedures to approximate this idealized estimate of the evolutionary history of *Desmognathus*, taking care to highlight potential areas of continuing uncertainty and foci for future research.

Integrating all these analyses in the context of delimiting terminal taxa requires some care. While we are overall very cautious in interpreting phylogenetic topologies given the apparent prevalence of gene flow and hypothesized impacts thereon, we treat reciprocal monophyly of geographically distinct clades as the clearest evidence for valid candidate species. We previously recognized 49 of these (Pyron et al., 2020). Based on our phylogenetic analyses (see below), we first determine whether any of our previous candidate species should be combined based on paraphyly revealed by additional sampling. If any geographically distinct clusters are supported by clustering analyses and are reciprocally monophyletic, we recognize them as new candidate species. Similarly, if any previously recognized candidate species are not distinguished by the population‐genetic analyses, we lumped them. Finally, if the delimitation analyses reveal significant genetic clusters that are not reciprocally monophyletic and exhibit significant hybridization and spatial genetic clines, we treat them as phylogeographic lineages within candidate species.

### 2.4 Phylogenetic inference

We estimated 233 individual gene trees using IQ‐TREE v2.1.3 (Minh, Schmidt, et al., 2020) with optimal models selected using ModelFinder (Kalyaanamoorthy et al., 2017) and support estimated using 1000 ultrafast bootstraps (Hoang et al., 2017) and the SHL‐aLRT branch statistic (Anisimova et al., 2011). We then estimated a concatenated phylogeny using partitioned models (Chernomor et al., 2016) under the optimal merging strategy for the 233 loci combined, also with UFBoot and SHL‐aLRT support values. For this topology, we finally estimated gene‐ and site‐concordance factors (gCF/sCF) from the individual locus alignments (Minh et al., 2020). We conducted five runs and used the best tree as the starting point for a final analysis.

We initially evaluated estimating a species tree under the assumptions of the multi‐species coalescent (MSC) model assuming incomplete lineage‐sorting (ILS) as the primary driver of gene‐tree discordance, in the program ASTRAL‐III v5.7.7 (Zhang et al., 2018), which has shown overall high accuracy in simulation (Chou et al., 2015). However, numerous recent authors have cast doubt on the accuracy of these methods in the face of extensive gene flow (Jiao et al., 2020; Leaché et al., 2014; Solís‐Lemus & Ané, 2016), as we observe in our dataset. Preliminary analyses of this dataset using all specimens and loci yielded anomalous topologies with low support that were also incongruent with species‐tree results from our previous study sampling many of the same specimens and loci (Pyron et al., 2020). As we were unable to address the potential confounding effects of ILS and gene flow on MSC‐based species trees, we proceeded with the concatenated and gene‐tree estimates alone.

### 2.5 Clustering and admixture analyses

The initial naming of *Desmognathus* clades was primarily qualitative (Kozak et al., 2005), giving a letter designation to monophyletic sublineages of existing morphospecies using a tree‐based procedure (Wiens & Penkrot, 2002). Later researchers attempted to formalize this nomenclature with systematic ‘ecodrainage’ sampling to ensure that all relevant potential lineages and geographic genetic segments were sampled (Beamer & Lamb, 2020). Our subsequent designation of 49 mito‐nuclear candidate species was based primarily on qualitative geographic and topological assignment to these clades with relatively limited sampling of populations (Pyron et al., 2020). Thus, the population‐level validity of these taxa remains unknown.

**FIGURE 3:**
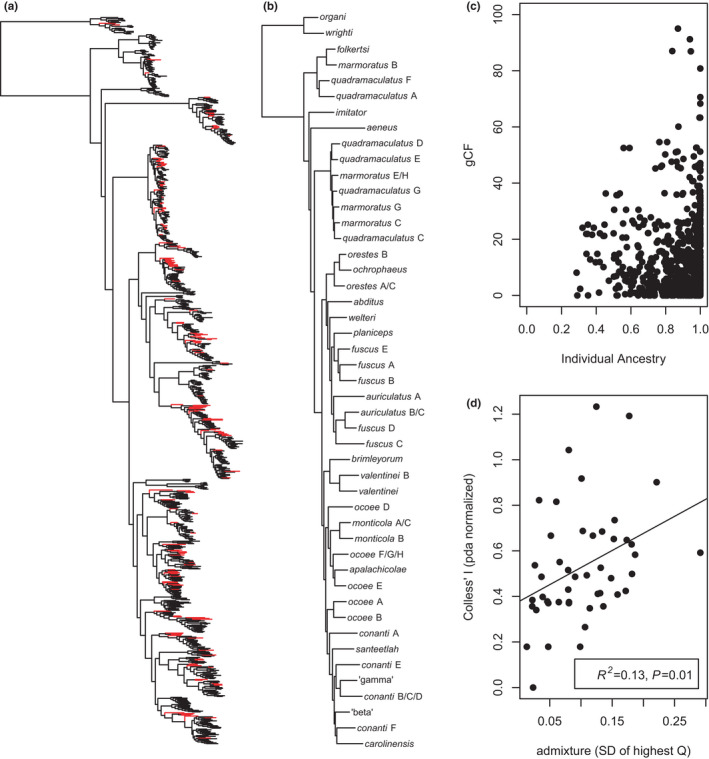
Concatenated ML estimate of 233 AHE genes for 894 specimens with highly admixed specimens (maximum individual ancestry ≤0.8) highlighted in red, typically occupying early‐diverging positions on long terminal branches (a) and reduced to the 47 distinct candidate species (b). The gCF support values for the placement of terminal species appear to be constrained by their maximum individual ancestry (c), and variance in ancestry is strongly related to topological imbalance of the candidate species phylogeny (d), where higher variance in maximum individual ancestry coefficients indicates clades exhibiting more hybridization

To provide a robust quantitative basis for future species‐delimitation analyses based on integrative datasets, including morphology, ecology, etc., we performed several clustering and admixture analyses to assign individuals to quantitatively identified candidate species. For manageability, we first divided the concatenated topology into 12 groups of mito‐nuclear candidate species as defined in our previous analyses (Figure 3; Table 1). We extracted SNPs with <20% missing data from each locus, removed singletons (Linck & Battey, 2019), and concatenated them into clade‐specific matrices. We first visualized nucleotide diversity relative to apparent genetic clusters using a PCA plot of the SNP matrix for those specimens (Dray & Dufour, 2007) in the R package ‘adegenet’ (Jombart, 2008), with individuals coded by their previously assigned mito‐nuclear candidate species.

**TABLE 1:** List of 47 candidate species of *Desmognathus* delimited using the phylogenomic dataset and phylogenetic and clustering analyses presented here, along with 30 estimated phylogeographic lineages within 13 candidate species for a total of 64 geographic genetic units, and reference to the results figure for each

Clade	Candidate	Lineage	Figure
Pigmy	*organi*	–	Figure 4
	*wrighti*	*wrighti* A1	Figure 4
	–	*wrighti* A2	Figure 4
Nantahala	*folkertsi*	–	Figure 6
	*marmoratus* B	–	Figure 6
	*quadramaculatus* F	–	Figure 6
	*quadramaculatus* A	*quadramaculatus* A1	Figure 5
	–	*quadramaculatus* A2	Figure 5
Seepage	*imitator*	–	Figure 7
	*aeneus*	*aeneus* A1	Figure 7
	–	*aeneus* A2	Figure 7
	–	*aeneus* A3	Figure 7
	–	*aeneus* A4	Figure 7
Pisgah	*quadramaculatus* D	–	Figure 9
	*quadramaculatus* E	*quadramaculatus* E1	Figure 8
	–	*quadramaculatus* E2	Figure 8
	*marmoratus* E/H	–	Figure 9
	*quadramaculatus* G	–	Figure 9
	*marmoratus* G	–	Figure 9
	*marmoratus* C	–	Figure 9
	*quadramaculatus* C	–	Figure 9
Appalachian	*orestes* B	–	Figure 11
	*ochrophaeus*	–	Figure 10
	*orestes* A/C	*orestes* A	Figure 11
	–	*orestes* C	Figure 11
Cumberland	*abditus*	–	Figure 12
	*welteri*	–	Figure 12
Upland *fuscus*	*planiceps*	–	Figure 14
	*fuscus* E	–	Figure 14
	*fuscus* A	–	Figure 14
	*fuscus* B	*fuscus* B1	Figure 13
	–	*fuscus* B2	Figure 13
	–	*fuscus* B3	Figure 14
Lowland *fuscus*	*auriculatus* A	–	Figure 15
	*auriculatus* B/C	*auriculatus* B	Figure 15
	–	*auriculatus* C	Figure 15
	*fuscus* D	–	Figure 16
	*fuscus* C	*fuscus* C1	Figure 16
	–	*fuscus* C2	Figure 16
	–	*fuscus* C3	Figure 16
Ouachita	*brimleyorum*	–	Figure 17
	*valentinei* B	–	Figure 17
	*valentinei* A	–	Figure 17
*ocoee*	*ocoee* D	*ocoee* D1	Figure 19
	–	*ocoee* D2	Figure 19
	*monticola* A/C	–	Figure 18
	*monticola* B	–	Figure 18
	*ocoee* F/G/H	–	Figure 19
	*apalachicolae*	*apalachicolae* A1	Figure 19
	–	*apalachicolae* A2	Figure 19
	*ocoee* E	*ocoee* E1	Figure 19
	–	*ocoee* E2	Figure 19
Balsam	*ocoee* A	–	Figure 20
	*ocoee* B	–	Figure 20
*conanti*	*conanti* A	*conanti* A1	Figure 22
	–	*conanti* A2	Figure 22
	*santeetlah*	–	Figure 22
	*conanti* E	–	Figure 21
	‘gamma’	–	Figure 22
	*conanti* B/C/D	*conanti* B/D	Figure 21
	–	*conanti* C	Figure 21
	‘beta’	–	Figure 22
	*conanti* F	–	Figure 22
	*carolinensis*	–	Figure 22

Notes

These taxa and lineages circumscribe the genetic diversity of all known, extant population segments within the genus. For the history of these naming conventions and previous mitochondrial and nuclear estimates of these candidate species, see Beamer and Lamb (2020), Kozak et al. (2005) and Pyron et al. (2020).

We initially evaluated the use of DAPC (Jombart et al., 2010) to identify statistical clusters. However, the results were identical to our clade‐level admixture analyses (see below) in all but three cases; *aeneus* and *fuscus* where DAPC estimated additional phylogeographic structure, and *marmoratus* G, where the two disagreed on hybrid assignment to a parental species. We believe these minor differences to be a result of DAPC’s failure to account for gene flow, which previous authors suggest overestimates diversity and mis‐specifies hybrids (Chan et al., 2017), and we therefore do not present these results. We also initially investigated the ‘snapclust’ algorithm (Beugin et al., 2018), which identifies the number of *K* clusters in Hardy–Weinberg Equilibrium, and can identify F_1_ and F_2_ hybrids when *K* = 2. However, preliminary results were apparently anomalous for many clades, likely due to the strong violation of the Hardy–Weinberg assumption of no migration, and we did not pursue this approach further.

Our final aim is to estimate the prevalence and location of hybrid individuals, and the overall degree and spatial extent of genomic admixture between candidate species. We approached this with both individual‐ and taxon‐based approaches. First, we used the ‘sNMF’ algorithm in the R package ‘LEA’ to estimate individual ancestry coefficients for each specimen (Frichot et al., 2014). For each clade or set of comparisons, we first optimized the regularization parameter *α* for values spanning several orders of magnitude: 1, 5, 10, 50, 100, 500, and 1000. Frichot et al. (2014) initially tested values up to 10,000 but found that values above 1000 were generally discarded for most datasets by the cross‐entropy criterion. We selected the value of *α* that minimized median cross‐entropy across 100 replicates. Using the optimal value of *α* for each clade, we then estimated ancestry using the values of *K* derived from the clustering analyses for that clade (see above) as well as determining the optimal value of *K* minimizing median cross‐entropy across 100 replicates, if these differed. In a few cases where an elbow did not form, we selected the lowest value of *K* representing a significant improvement in cross‐entropy using the ‘notch’ test of the boxplots (i.e., overlapping 95% SE of the median).

Second, we estimated gene flow across mito‐nuclear candidate species boundaries using the Patterson's *D* and *f*_4_‐ratio statistics in the package ‘Dsuite’ (Malinsky et al., 2021). These branch‐based approaches estimate hybrid ancestry on a given topology, inferred from the expected frequency distributions of site patterns under ILS versus reticulation. Based on the topological evaluation of candidate‐species monophyly in the concatenated phylogeny supporting 47 distinct lineages, we condensed the 894‐taxon topology to these 47 clades. As Dsuite requires an outgroup, we used the “pigmy” clade of *organi* + *wrighti*, since the early‐diverging position of this lineage was not in dispute, nor did we expect it to be involved in hybridization events with other *Desmognathus*. We used this topology as the input for Dtrios, Fbranch, and for plotting results, yielding a comparison of the 46 ‘ingroup’ candidate species.

Crucially, this implementation can handle large numbers of species, integrating over all 4‐taxon subtrees from a given phylogeny. This allows for the inference of multiple hybridization events, potentially including scenarios representing non‐level‐1 networks. Whether these inferences can somehow bypass the issues of topological non‐identifiability of such networks remains unclear. Additionally, Dsuite can only directly infer tip‐to‐tip and branch‐to‐tip events; we estimate several instances where deep‐time branch‐to‐branch reticulations appear to be reflected across numerous significant branch‐to‐tip events (see below). Accurately identifying such models is still challenging, and similar distributions of allele frequencies can be generated by a variety of processes, such as variation in substitution rates (Pease & Hahn, 2015), ancestral population structure (Eriksson & Manica, 2012), and ghost admixture (Lawson et al., 2018).

### 2.6 Hybridization and topology

Recent authors (Chan et al., 2022; Dolinay et al., 2021) have provided verbal models and preliminary empirical evidence for an intuitive process: the presence of hybrid individuals in a phylogenetic analysis of phylogeographic datasets seems to create artifactual topologies. Specifically, hybrids between two parental populations seem to form ladder‐like grades between them in proportion to their individual ancestry from each. They may also attract other hybrid individuals with similar admixture profiles, creating false clades that appear to represent real, distinct evolutionary lineages that are merely statistical clusters of hybrids. Such profiles can result from a wide variety of unrelated processes (Lawson et al., 2018). We noted the possible presence of these artifacts in our preliminary analyses. Specifically, we noticed highly imbalanced clades with hybrid specimens in early‐diverging positions.

We assess the presence of these potential artifacts in two ways. First, we tested for a relationship between the gCF supporting the placement of each terminal specimen and the largest individual ancestry coefficient for that specimen. Presumably, highly admixed specimens cannot, by definition, be supported by high gCF. This approach duplicates some gCF values for sister pairs of specimens, but our null hypothesis is that hybrids (i.e., high‐admixture specimens) will cluster together, which would preserve the expected pattern. Second, we tested whether the imbalance of the subtrees for each of the 47 mito‐nuclear candidate species (except *marmoratus* G which only had a single specimen) was related to the amount of hybridization within that clade. We measured imbalance as Colless’ *I*, normalized proportional to distinguishable arrangements, rather than a Yule process which is likely inappropriate at the phylogeographic level (Blum & François, 2006). We measured clade‐level hybridization as the standard deviation (SD) of maximum individual ancestry, equal to 0 if all individuals were pure parentals. Conceivably, the SD could also be 0 if all specimens had the exact same hybrid ancestry, but this would require that the entire lineage be composed of identical hybrids, which is unlikely; all clades contained at least some pure parentals, making this an appropriate measure.

## 3 RESULTS

### 3.1 Phylogenetic inference

**FIGURE 4:**
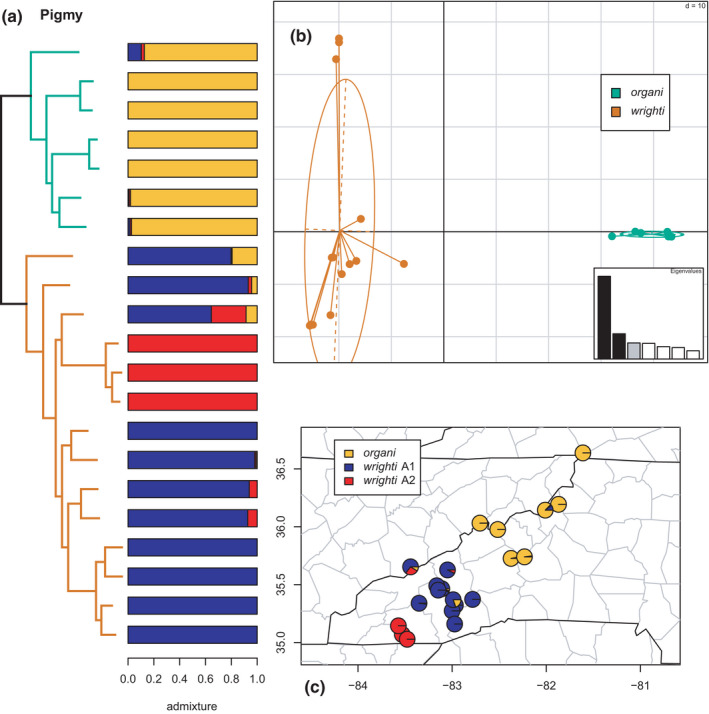
Phylogeny and barplot (a), PCA (b), and map (c) of the pigmy clade (*organi* + *wrighti*), with individual ancestry coefficients from estimated phylogeographic lineages. The colors on the tree branches and PCA correspond to the mito‐nuclear candidate species, while those of the barplot and map correspond to the phylogeographic lineages inferred by sNMF. This pattern is consistent across figures, but the colors are recycled for each clade

**FIGURE 5:**
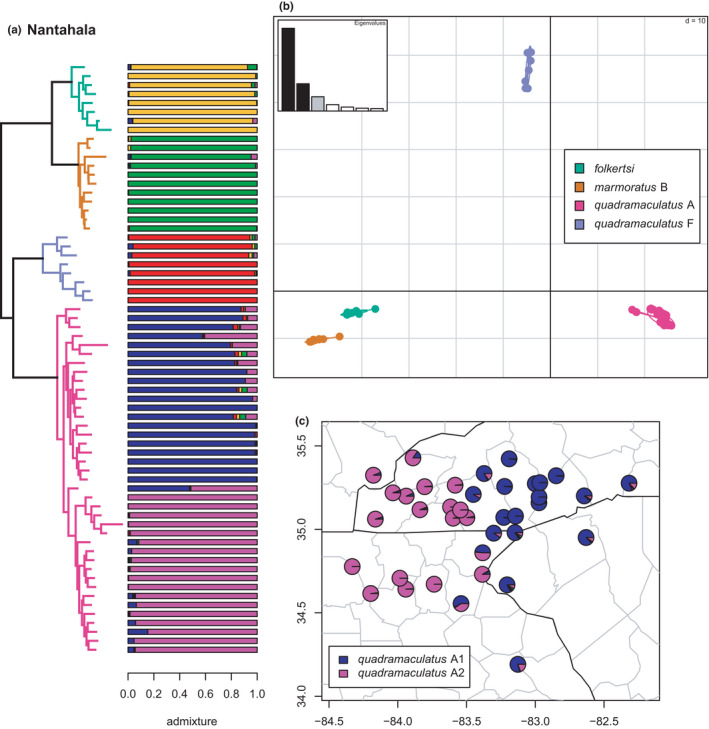
Phylogeny of the Nantahala clade (*folkertsi*, *marmoratus* B, *quadramaculatus* F, and *quadramaculatus* A) with branches (a) and PCA (b) colored by mito‐nuclear candidate species, along with barplot and map of estimated individual ancestry coefficients (c) for *quadramaculatus* A1/A2 colored by inferred phylogeographic lineages from the sNMF admixture analysis

**FIGURE 6:**
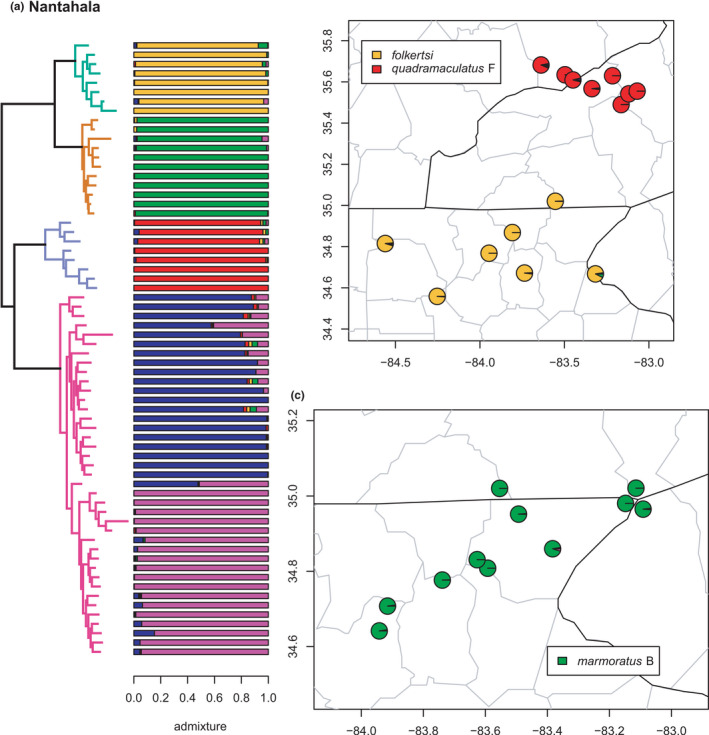
Phylogeny of the Nantahala clade (*folkertsi*, *marmoratus* B, *quadramaculatus* F, and *quadramaculatus* A) with branches (a) colored by mito‐nuclear candidate species, along with barplot and map of estimated individual ancestry coefficients (b) for *quadramaculatus* F and *folkertsi*, and (c) for *marmoratus* B

**FIGURE 7:**
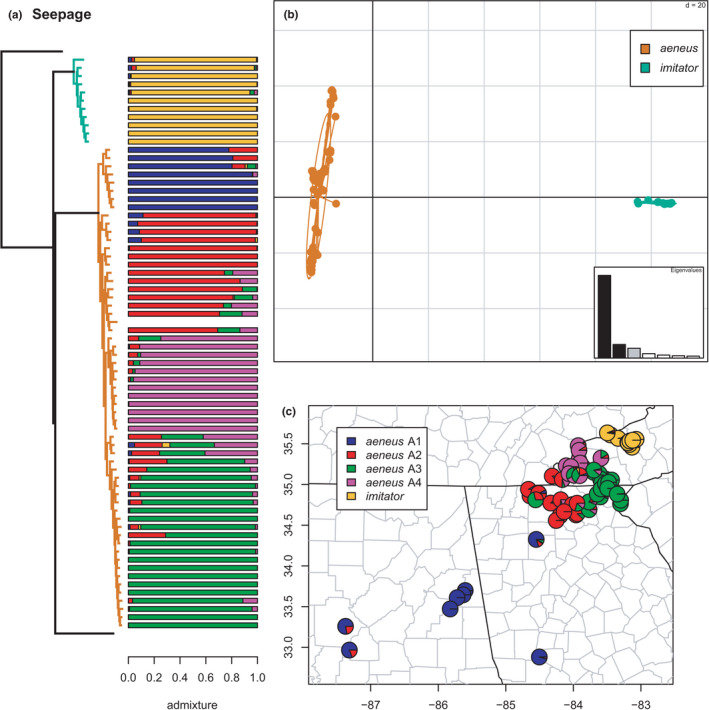
Phylogeny of the Seepage clade (*aeneus* and *imitator*) with branches (a) and PCA (b) colored by mito‐nuclear candidate species, along with barplot and map of estimated individual ancestry coefficients (c) for each candidate species colored by inferred phylogeographic lineages from sNMF admixture analysis. The horizontal blank in the barplot was a sample dropped from the clustering and admixture analyses due to missing data

**FIGURE 8:**
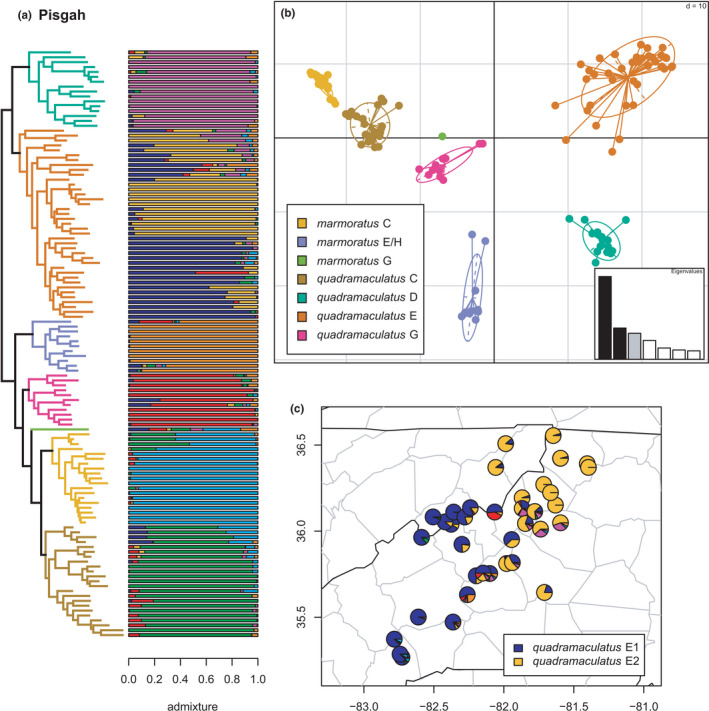
Phylogeny of the Pisgah clade (*quadramaculatus* D, *quadramaculatus* E, *marmoratus* E/H, *quadramaculatus* G, *marmoratus* G, *marmoratus* C, and *quadramaculatus* C) with branches (a) and PCA (b) colored by mito‐nuclear candidate species, along with barplot and map of estimated individual ancestry coefficients (c) for *quadramaculatus* E colored by inferred phylogeographic lineages from sNMF admixture analysis

**FIGURE 9:**
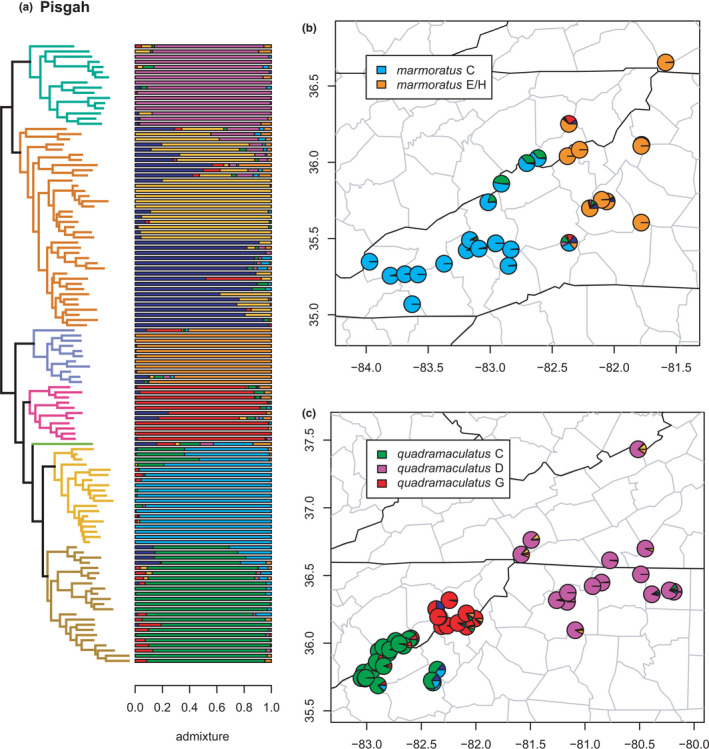
Pisgah clade (*quadramaculatus* D, *quadramaculatus* E, *marmoratus* E/H, *quadramaculatus* G, *marmoratus* G, *marmoratus* C, and *quadramaculatus* C) with branches (a) colored by mito‐nuclear candidate species, along with barplot and map of estimated individual ancestry coefficients (b) for *marmoratus* E/H and C, and (c) for *quadramaculatus* C, D, and G

**FIGURE 10:**
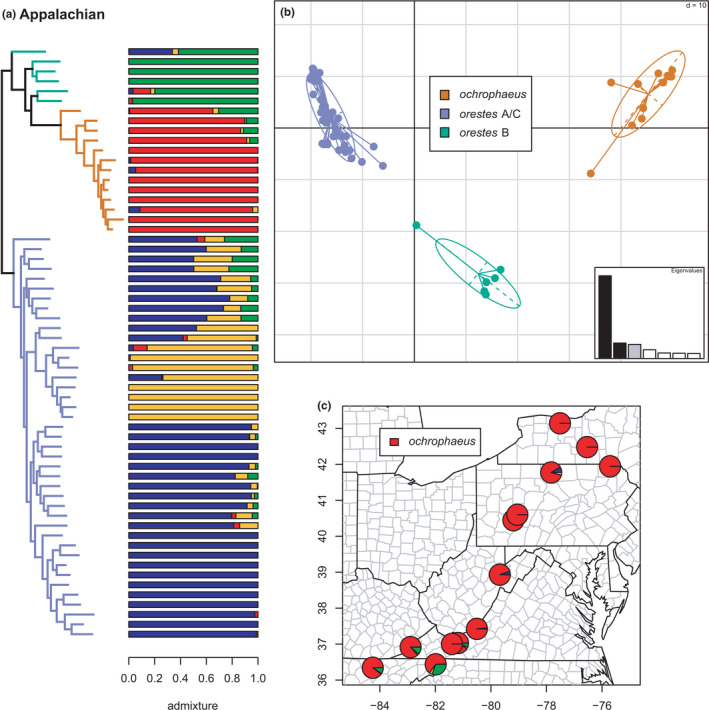
Phylogeny of the Appalachian clade (*orestes* B, *ochrophaeus*, and *orestes* A/C) with branches (a) and PCA (b) colored by mito‐nuclear candidate species, along with barplot and map of estimated individual ancestry coefficients (c) for *ochrophaeus*

**FIGURE 11:**
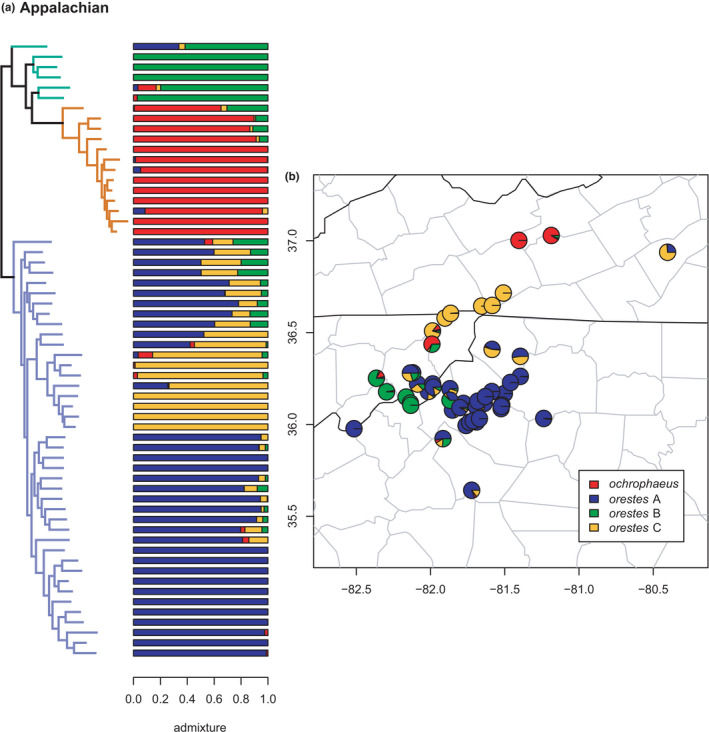
Phylogeny of the Appalachian clade (*orestes* B, *ochrophaeus*, and *orestes* A/C) with branches (a) colored by mito‐nuclear candidate species, along with barplot and map of estimated individual ancestry coefficients (b) for all lineages, focusing on *orestes* B and A/C

**FIGURE 12:**
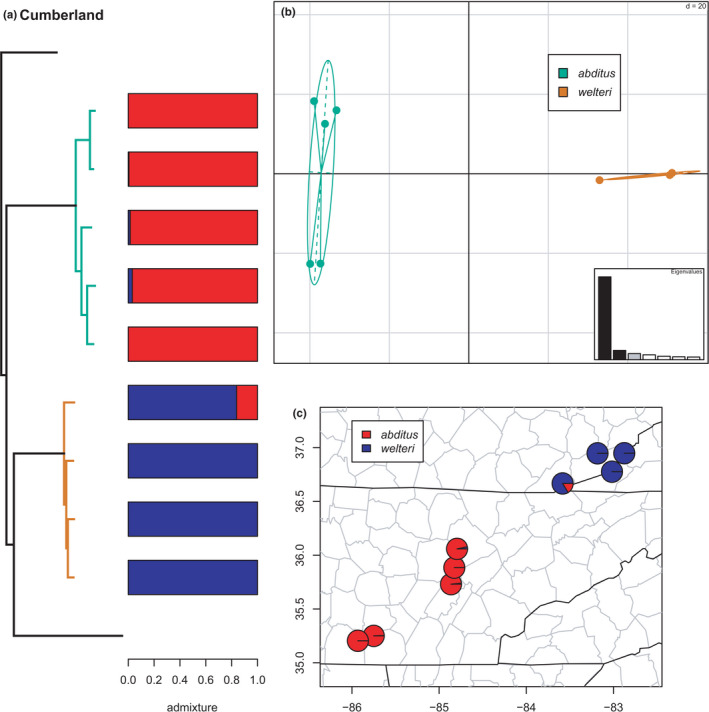
Phylogeny of the Cumberland clade (*abditus* and *welteri*) with branches (a) and PCA (b) colored by mito‐nuclear candidate species, along with barplot and map of estimated individual ancestry coefficients (c) for each candidate species colored by inferred lineages from sNMF admixture analysis

**FIGURE 13:**
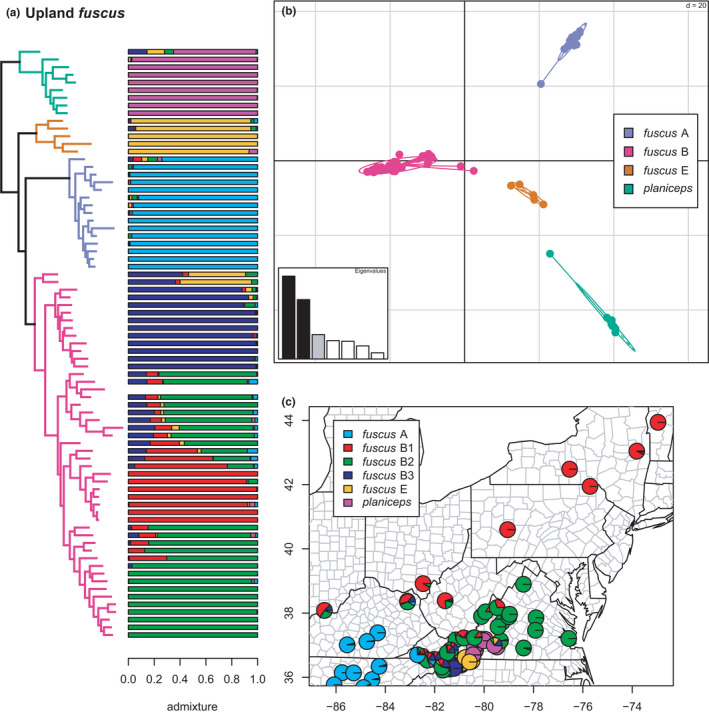
Upland *fuscus* clade (*planiceps*, *fuscus* E, A, and B) with branches (a) colored by mito‐nuclear candidate species, along with barplot and map of estimated individual ancestry coefficients (b) focused on *fuscus* B, E, and *planiceps*. The blank bar in the vertical plot was a sample dropped due to missing data from the clustering and admixture analyses

**FIGURE 14:**
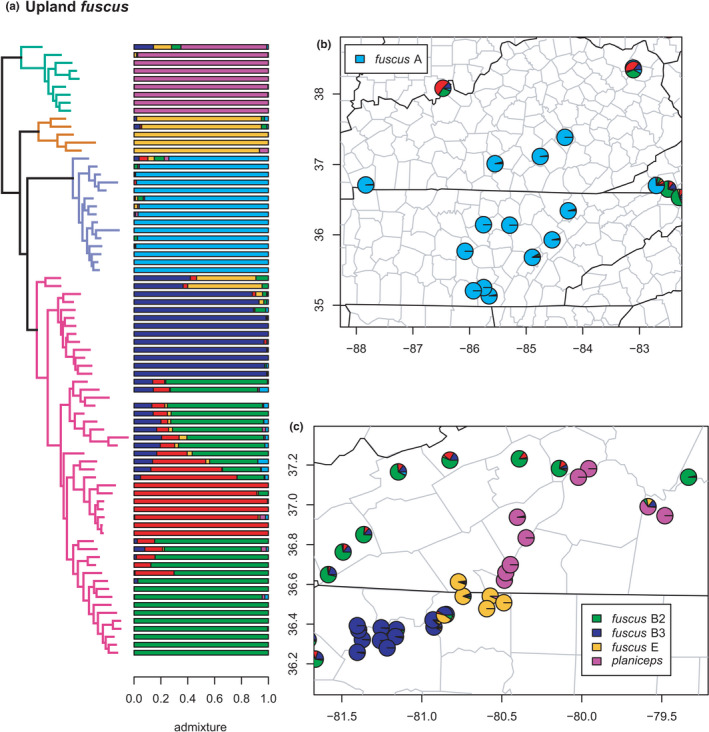
Upland *fuscus* clade (*planiceps*, *fuscus* E, A, and B) with branches (a) colored by mito‐nuclear candidate species, along with barplot and map of estimated individual ancestry coefficients focused on (b) *fuscus* A, and (c) *fuscus* E and *planiceps*. The blank bar in the vertical plot was a sample dropped due to missing data from the clustering and admixture analyses

**FIGURE 15:**
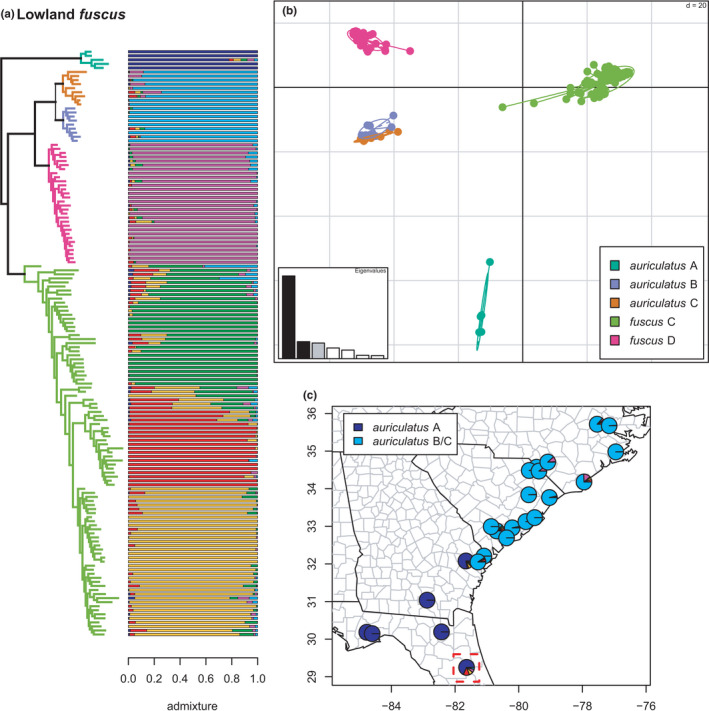
Lowland fuscus clade (auriculatus A, B, and C; fuscus C and D) with branches and PCA (a) colored by mito‐nuclear candidate species, along with barplot and map of estimated individual ancestry coefficients (c) focused on auriculatus A and B/C. The dotted red outline on the map highlights the formalin‐fixed specimens (USNM 468094‐5). That pie chart shows the mean of their estimated individual ancestry coefficients from the two sNMF runs on subsetted SNP matrices. They are thus not, strictly speaking, equivalent, but the full and reduced analyses all recovered the same six lineages, and we, therefore, present them here for visual comparison. The full results for these specimens (see below), including the reduced phylogeny, are given in the SI

**FIGURE 16:**
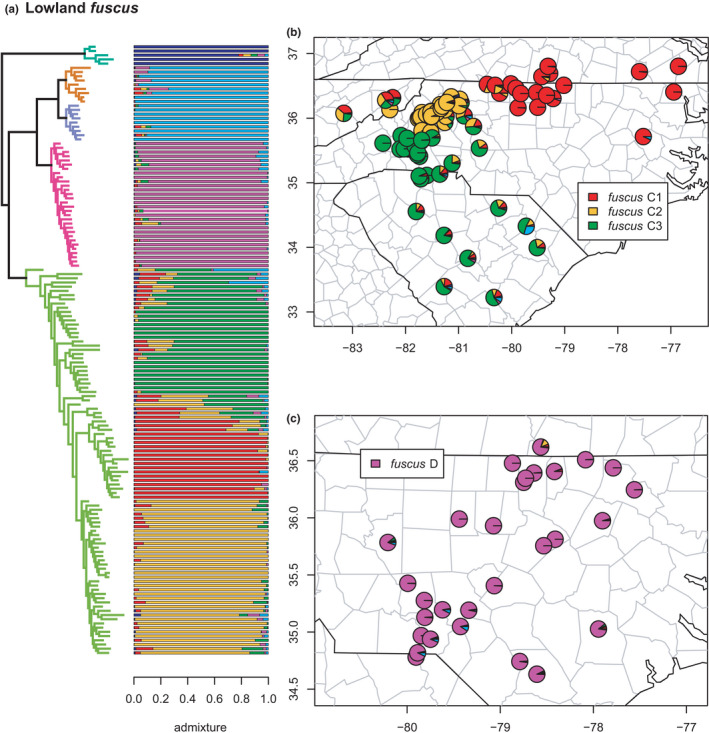
Lowland *fuscus* clade (*auriculatus* A, B, and C; *fuscus* C and D) with branches (a) colored by mito‐nuclear candidate species, along with barplot and map of estimated individual ancestry coefficients focused on (b) *fuscus* C, and (c) *fuscus* D

**FIGURE 17:**
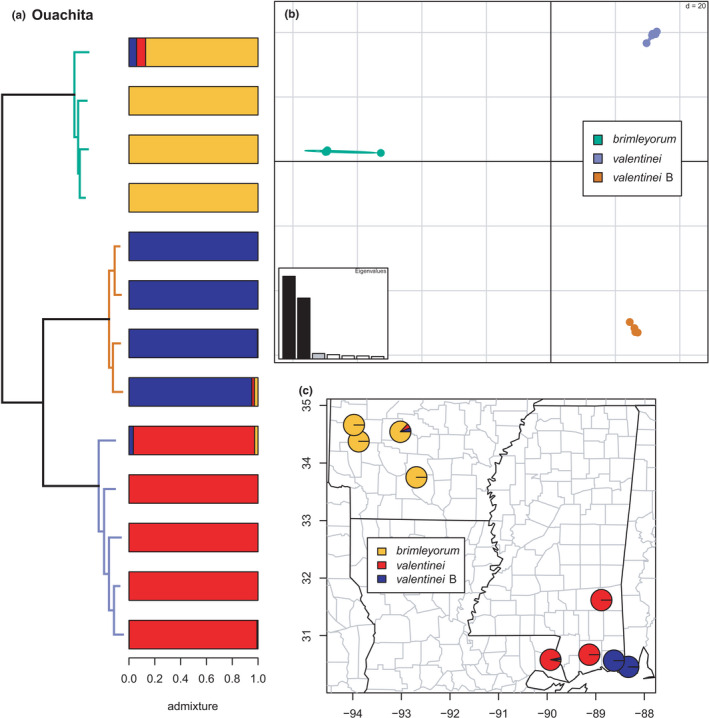
Phylogeny of the Ouachita clade (*brimleyorum*, *valentinei*, and *valentinei* B) with branches (a) and PCA (b) colored by mito‐nuclear candidate species, along with barplot and map of estimated individual ancestry coefficients (c) for each candidate species

**FIGURE 18:**
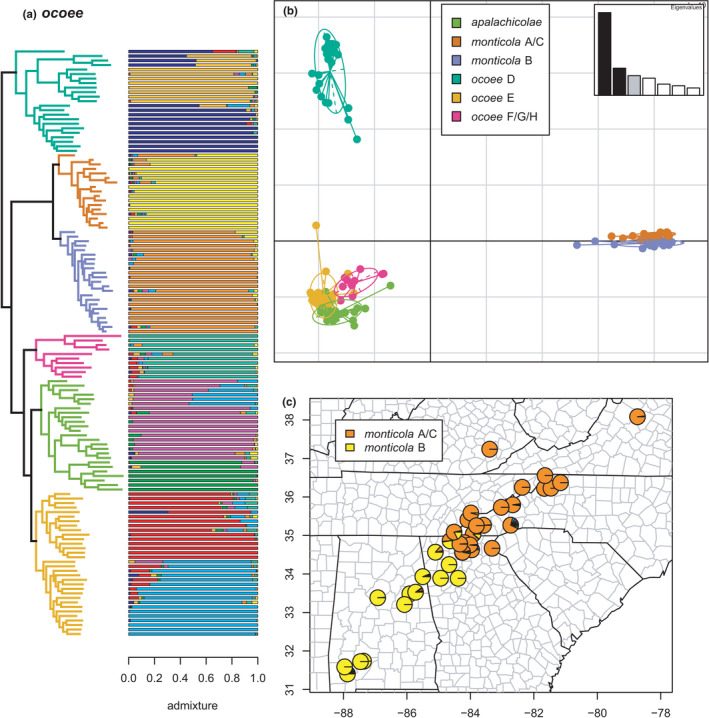
*ocoee* clade (*ocoee* D, *monticola* A/C & B, *ocoee* F/G/H, *apalachicolae*, and *ocoee* E) with branches (a) and PCA (b) colored by mito‐nuclear candidate species, along with barplot and map of estimated individual ancestry coefficients (c) focused on *monticola* A/C and B

**FIGURE 19:**
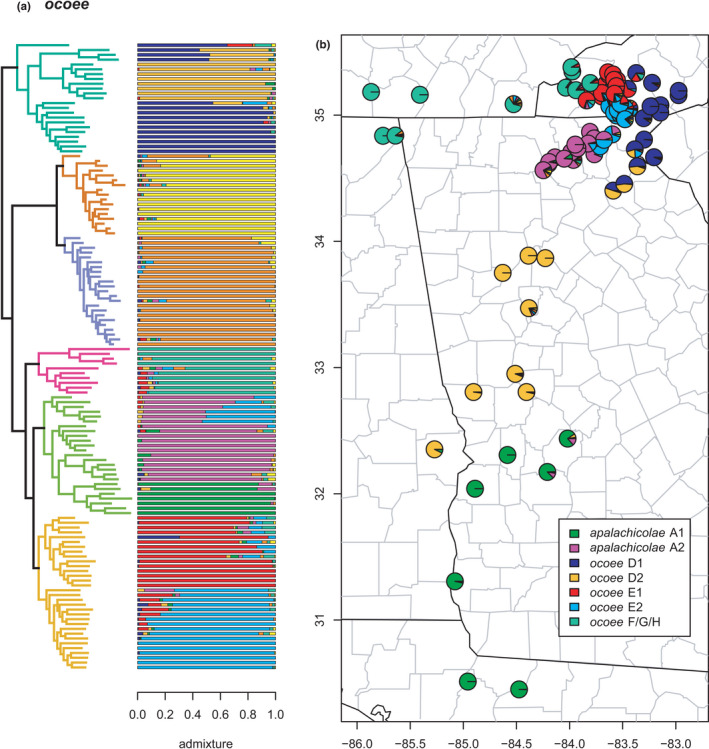
*ocoee* clade (*ocoee* D, *monticola* A/C & B, *ocoee* F/G/H, *apalachicolae*, and *ocoee* E) with branches (a) colored by mito‐nuclear candidate species, along with barplot and map of estimated individual ancestry coefficients (b) focused on *ocoee* D, E, F/G/H, and *apalachicolae*

**FIGURE 20:**
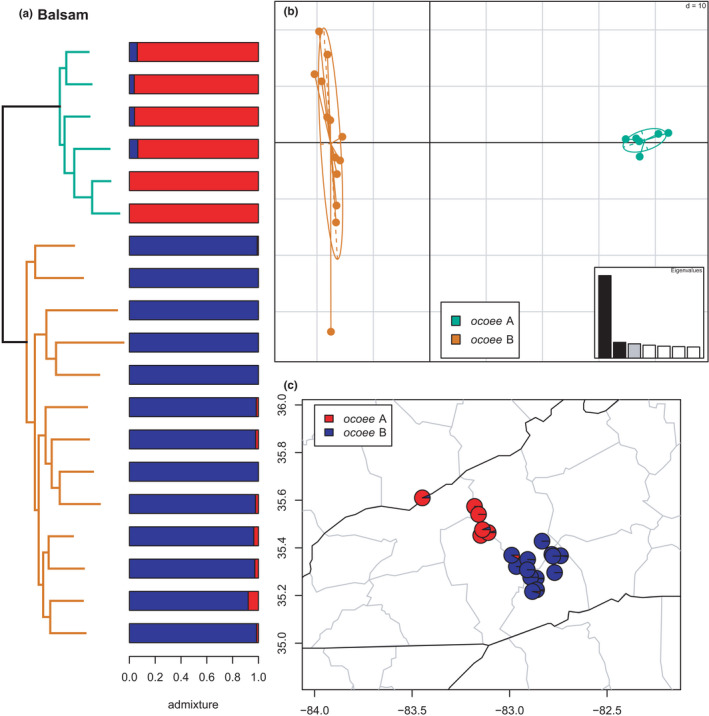
Phylogeny of the Balsam clade (*ocoee* A & B) with branches (a) and PCA (b) colored by mito‐nuclear candidate species, along with barplot and map of estimated individual ancestry coefficients (c) colored by inferred lineages from the sNMF admixture analysis

**FIGURE 21:**
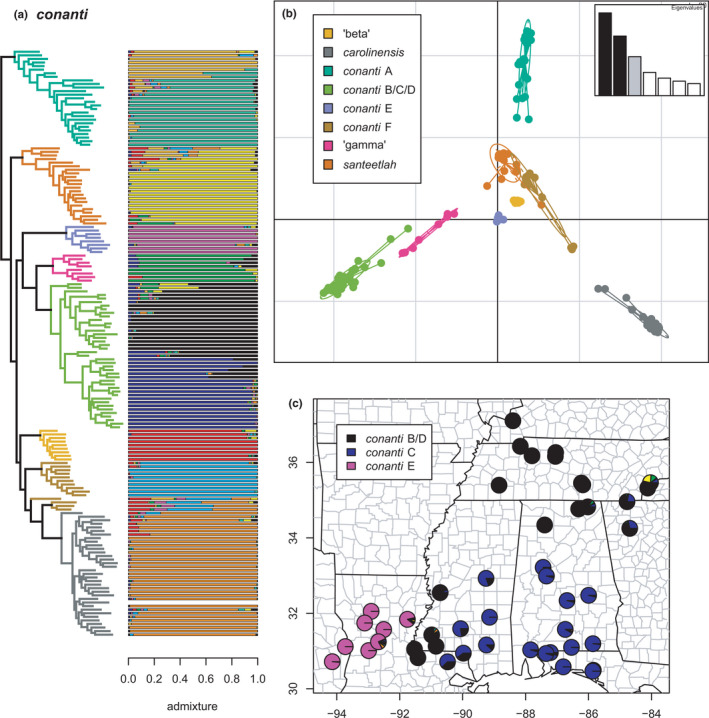
The *conanti* species group (*conanti* A, *santeetlah*, *conanti* E, ‘gamma,’ *conanti* B/C/D, ‘beta,’ *conanti* F, and *carolinensis*) with branches (a) and PCA (b) colored by mito‐nuclear candidate species, along with barplot and map of estimated individual ancestry coefficients (c) focused on *conanti* B/D, C, and E

**FIGURE 22:**
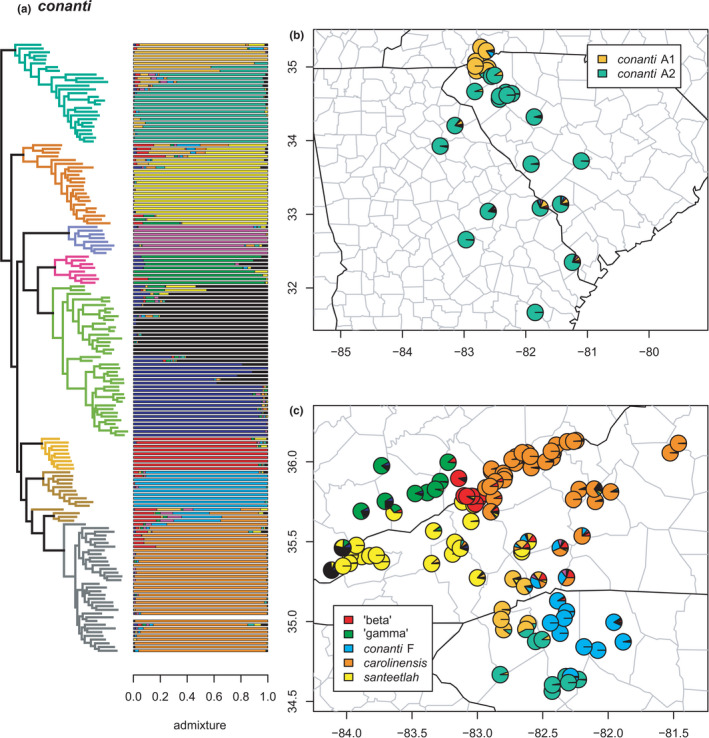
The conanti species group (conanti A, santeetlah, conanti E, ‘gamma,’ conanti B/C/D, ‘beta,’ conanti F, and carolinensis) with branches (a) colored by mito‐nuclear candidate species, along with barplot and map of estimated individual ancestry coefficients (b) focused on conanti A and (c) ‘beta,’ carolinensis, conanti F, santeetlah, and ‘gamma’

**FIGURE 23:**
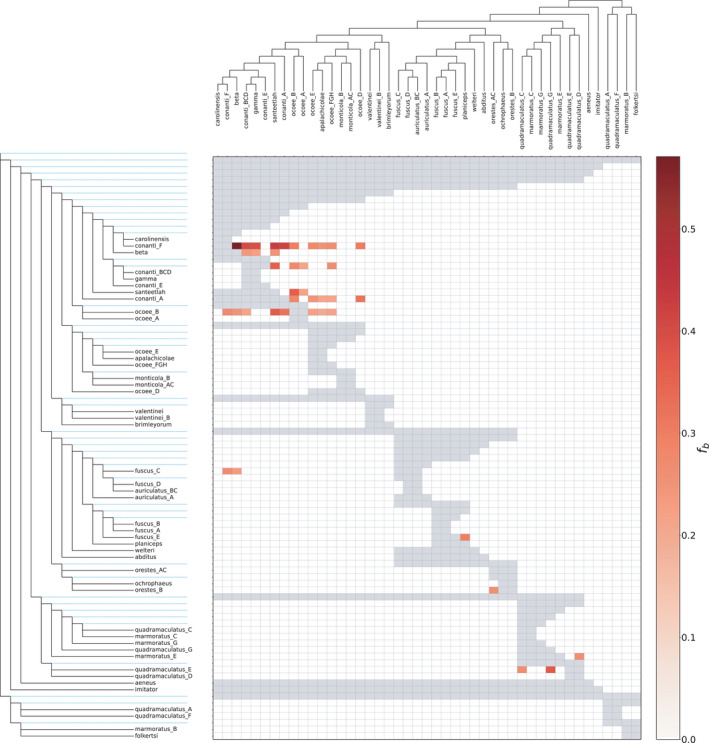
Matrix of *f*_b_ values from the Dsuite analysis of 233 AHE loci, using the concatenated ML topology for the 47 ingroup candidate species, treating the “pigmy” clade (*organi* + *wrighti*) as the outgroup. These values represent the proportion of alleles shared between the donor (column) and recipient (row) branches in excess of that predicted by the MSC model, indicating likely instances of introgression. Values have been truncated at a significance level of 20%. This result suggests hybridization between multiple candidate species in various *conanti*, *fuscus*, *marmoratus*, *quadramaculatus*, *ocoee*, and *orestes* lineages

The concatenated estimate of the 894‐taxon dataset is generally similar to recent results (Beamer & Lamb, 2020; Kozak et al., 2005; Pyron et al., 2020; Weaver et al., 2020), with no major topological novelties or newly discovered clades (Figures 3, 4, 5, 6, 7, 8, 9, 10, 11, 12, 13, 14, 15, 16, 17, 18, 19, 20, 21, 22, 23). The one remarkable difference is the placement of *carolinensis*, which is nested within the *conanti* species group (Figure 3a,b), a placement that has not been recovered in previous studies. This placement is strongly supported (~100%) by BS and SHL, but weakly supported by gCF (~0%) and sCF (~33%), suggesting strongly conflicting signal among genes (Minh, Hahn, et al., 2020). This is potentially related to ILS, hybridization, or gene‐tree error, though we discard the latter explanation given the length and informativeness of our loci. It is difficult to untangle ILS from gene flow (Wang et al., 2018; Zhou et al., 2017), but given the prevalence of admixture, we are confident that gene flow is chief among the patterns driving topological variation across the many recent estimates. This hypothesis corroborates our previous results (Pyron et al., 2020).

Cross‐referencing the topology with our admixture estimates (see below) suggests that numerous hybrid individuals occupy intermediate positions as an artifactual consequence of their admixed ancestry, which cannot be resolved adequately by concatenation or species‐tree analysis. We estimate that 169 of the 894 specimens have >20% ancestry from a secondary phylogeographic lineage or candidate species (Figure 1a). Chief among these include specimens of the Pisgah (Figure 8), Appalachian (Figure 11), and *conanti* (Figure 22) clades (Table 1). Overall, none of the 49 mito‐nuclear candidate species appear to be artifactual clades composed entirely of hybrids. However, a group of four heavily admixed specimens assigned to *conanti* F from southwestern North Carolina do appear to form such a cluster, estimated as the sister lineage to *carolinensis* in the concatenated tree (Figure 22). Additionally, the *marmoratus* G lineage from the Pisgah clade may also represent such a hybrid (Figure 9).

Regarding our preliminary assessment of the impact of hybridization on the topological placement of admixed individuals, we find some support for the conclusions of Chan et al. (2022) and Dolinay et al. (2021). A large proportion of the 169 admixed specimens are concentrated in ladder‐like topological positions or on long terminal branches (Figure 3a). Similarly, there is a triangular (constraining) relationship between individual ancestry and gCF support for placement (Figure 3c); higher gCF is only observed for individuals with greater ancestry from single populations, and admixture limits the maximum observed gCF. Finally, there is a significant relationship (*R* ^2^ = .13, *p* = .01) between clade level admixture (SD of individual ancestry coefficients *Q*) and Colless’ *I* normalized by the PDA model (Figure 3d). Thus, clades with more admixed individuals are more imbalanced on average.

### 3.2 Candidate species and phylogeographic lineages

There are four major differences between our results and previous studies (Beamer & Lamb, 2020; Kozak et al., 2005; Pyron et al., 2020). First, *conanti* B/D & C are not reciprocally monophyletic and contain multiple admixed specimens with a geographically broad hybrid zone. We, therefore, collapsed them into a single candidate species *conanti* B/C/D, representing the nominotypical lineage encompassing the type locality (see Beamer & Lamb, 2020).

Second, specimens of *valentinei* from the southern Pascagoula and Escatawpa drainages of Mississippi and Alabama form a reciprocally monophyletic group exhibiting almost no gene flow with *valentinei*. Distinctiveness of this lineage was noted in previous analyses with more limited sampling (Beamer & Lamb, 2020; Means et al., 2017; Pyron et al., 2020). We, therefore, recognize it as *valentinei* B, a newly delimited mito‐nuclear candidate species.

Third, the population‐level analyses estimate *auriculatus* B & C as a single cluster; we collapse them to *auriculatus* B/C. Finally, while *marmoratus* G is estimated as a hybrid originating from multiple parental lineages, it possesses a unique mitochondrial haplotype (Beamer & Lamb, 2020) and topological position, and we, therefore, continue to recognize it as a provisionally distinct candidate species. Our overall analyses, thus, support 47 distinct candidate species based on population‐level analysis of phylogenomic data (Table 1).

Despite the stability of candidate species delimited from 2005 (Beamer & Lamb, 2020; Kozak et al., 2005; Pyron et al., 2020) to the present analyses, many exhibit admixture with other lineages, contain significant phylogeographic structuring, and show spatial genetic clines of gene flow between geographic clusters. Indeed, we estimate as many as 30 phylogeographic lineages within 13 of the 47 candidate species. Most of these exhibit admixture with respect to geographically, if not necessarily phylogenetically proximate groups. Following, we detail the results of the population‐level analyses with respect to the phylogenetic topology, spatial extent, and hybrid dynamics of the 64 lineages across the 12 clades in ascending phylogenetic order.

#### 3.2.1 Pigmy clade

As in most previous studies, *organi* and *wrighti* are monophyletic sister lineages in the phylogeny and supported as distinct by the clustering and admixture analyses (Figure 4). The sNMF results find an optimal *K* = 3, estimating geographic population structure within *wrighti* comprising a widespread phylogeographic lineage in the northern part of its range (Great Smoky and Great Balsam mountains) and a restricted lineage in the southern Nantahala mountains. We refer to these as *wrighti* A1 & A2, respectively. A small amount of admixture is estimated both between *wrighti* A1 & A2 and between *organi* and *wrighti* A1. Whether the latter represents ILS or possible genetic contact across the Asheville Basin as recently as the LGM is unclear (Crespi et al., 2003, 2010) and can be addressed with further geographic and genomic sampling in future studies. While the most proximate known populations of *organi* and *wrighti* are included here, other populations of *wrighti* are known from the western Smokies and surrounding mountains that were not sampled (Harrison, 2000).

#### 3.2.2 Nantahala clade

As in previous studies, *folkertsi*, *marmoratus* B, *quadramaculatus* A, and *quadramaculatus* F are monophyletic and supported as distinct by the phylogenetic and admixture analyses (Figures 5 and 6). Correspondingly, the first two and last two are sister lineages, together forming the Nantahala clade (Jones & Weisrock, 2018; Pyron et al., 2020). Selection of *K* by lowest median cross‐entropy yielded five clusters, corresponding to the four candidate species, one (*quadramaculatus* A) with two phylogeographic lineages. The sNMF analysis estimated the *quadramaculatus* A1 & A2 lineages east and west of the Little Tennessee River valley as in our previous study (Beamer & Lamb, 2020), with numerous admixed individuals in the hybrid zone associated with their contact. None of the candidate species show significant or notable evidence of gene flow (i.e., >10% individual ancestry) with any other geographically or phylogenetically proximate lineages.

#### 3.2.3 Seepage clade

As in previous studies, *aeneus* and *imitator* are reciprocally monophyletic species, which form either the successive outgroups to all other *Desmognathus* excluding the Pigmy (Beamer & Lamb, 2020; Kozak et al., 2005) or the Pigmy and Nantahala clades alone (Pyron et al., 2020), as here (Figure 3). Our previous species‐tree and network analyses of a smaller AHE dataset provided evidence that *aeneus* and *imitator* are, in fact, sister lineages (Pyron et al., 2020), but that a deep‐time reticulation involving the stem lineage of the *fuscus* and *conanti*‐group species is responsible for the poorly supported, non‐sister topologies recovered here and in previous mitochondrial and concatenated nuclear estimates (e.g., Weaver et al., 2020). The sNMF analyses yielded *K* = 5, recovering no geographic population structure within *imitator*, but four phylogeographic lineages within *aeneus* (Figure 7). This extensive intraspecific diversity was noted in previous analyses (Beamer & Lamb, 2020; Pyron et al., 2020), and corresponds roughly to different mountain segments in the southern Blue Ridge, with one lineage comprising Piedmont populations. The extensive parapatry and admixture of these lineages suggests a complex phylogeographic history that deserves further scrutiny.

#### 3.2.4 Pisgah clade

The Pisgah clade (see Jones & Weisrock, 2018; Pyron et al., 2020) represents a complex scenario of introgression and diversification that strains our definitions of phylogeographic lineages and candidate species (Figures 8 and 9). The complexity stems from three interrelated factors. First, the distinctiveness of the candidate species is supported by their formation of genealogically exclusive clades in the concatenated phylogeny. Second, they are also all morphologically diagnosable as either *marmoratus* or *quadramaculatus*, without any apparent morphological intermediacy, though lineages of these two morphospecies are interdigitated with each other and do not form monophyletic groups by phenotype (Beamer & Lamb, 2020; Jackson, 2005; Kozak et al., 2005; Pyron et al., 2020). Third, while all of them exhibit at least some “pure” individuals, each contains specimens with mixed genomic ancestry from other geographically or phylogenetically proximate lineages.

The seven previously defined candidate species are all reciprocally monophyletic; *marmoratus* C, E/H, and G, and *quadramaculatus* C, D, E, and G. These are generally supported by the clustering and admixture analyses with two major exceptions. Selection of *K* using minimum cross‐entropy did not form an elbow, but yielded *K* = 7 by the notch test. First, *marmoratus* G has a mixed (not unique) ancestry with roughly equal genomic contributions from the other six lineages, though a small plurality from *marmoratus* C (~30%). We observe significant admixture (i.e., at least ~20% individual ancestry) between *quadramaculatus* C & E, C & G, D & E, and E & G; between *marmoratus* E/H & *quadramaculatus* E; between *marmoratus* E/H & *quadramaculatus* G; and between *marmoratus* C & *quadramaculatus* C. Second, sNMF estimates two phylogeographic lineages *quadramaculatus* E1/E2, distributed approximately east and west of the boundary between the Upper French Broad/Nolichucky River drainages, with extensive hybridization between them. This region is also the contact zone between *carolinensis* and *orestes* (Tilley & Mahoney, 1996).

We also observe in the Pisgah clade a phenomenon that occurs here across the genus, where some specimens exhibit “streaks” of minor ancestry (<10%) from several other lineages that stack on the end of the barplot or occur together as a single cluster of slices in the pie chart. We generally do not interpret this further as evidence of admixture but note that it may have several distinct causes. First, it may represent actual evidence of small amounts of individual ancestry from those estimated clades. Second, it may represent noise in the algorithm or result from sequencing error, missing data, or ILS. Third, it may represent real individual ancestry from lineages not included in the comparison, such as other candidate species or ghost admixture from extinct lineages (Lawson et al., 2018). These patterns should be revisited in future studies with more extensive genomic sampling and targeted comparisons of candidate species. Additional genomic and geographic sampling is needed to clarify the origin and status of this complex radiation.

#### 3.2.5 Appalachian clade

Our results (Figures 10 and 11) for the *ochrophaeus* + *orestes* complex closely mirror most previous studies (Beamer & Lamb, 2020; Mead et al., 2001; Pyron et al., 2020; Tilley & Mahoney, 1996). The sNMF analyses estimated an optimal *K* = 4. The wide‐ranging candidate species *ochrophaeus* is monophyletic, distributed from Tennessee to eastern Canada. A paraphyletic group of specimens attributed to *orestes* B forms a clade with *ochrophaeus*, and this assemblage is the sister lineage of a monophyletic *orestes* A/C. The clustering and admixture analyses support these groups as distinct, with an additional phylogeographic separation of *orestes* A & C, though these are not monophyletic in the phylogeny. The ladder‐like phylogenetic grade purportedly caused by hybridization is on full display here, with highly admixed individuals occupying more early‐diverging topological positions. We observe significant admixture between *ochrophaeus* and *orestes* B, and between *orestes* B and *orestes* A/C, as in previous studies (Mead et al., 2001). The *orestes* A & C lineages also show extensive admixture along a broad zone in southwestern Virginia, eastern Tennessee, and northwestern North Carolina. Given the extensive and complex interplay of topography, mitochondrial exchange, and nuclear admixture in the region, additional genomic and geographic sampling is desirable to address the phylogeographic origins and taxonomic status of these populations, and of *orestes* B in particular.

#### 3.2.6 Cumberland clade

Both *abditus* and *welteri* form monophyletic candidate species in the phylogeny and are estimated as distinct (*K* = 2) by the admixture analyses in sNMF (Figure 12). The southwesternmost specimen of *welteri* from extreme southeastern Kentucky is estimated to have a small amount (~16%) of individual ancestry from *abditus*, but we do not consider this significant evidence of admixture between them at present. While we treat both candidate species together in this analysis, they are not sister lineages in the concatenated phylogenetic estimate presented here, but rather successive divergences (see also Weaver et al., 2020). However, they have been estimated as sister lineages in previous concatenated analyses, and in exploratory network analyses (Pyron et al., 2020; unpubl. data). In contrast, mitochondrial phylogenies estimate widely separated positions for the two taxa (Beamer & Lamb, 2020; Pyron et al., 2020). Current sampling is evidently inadequate for a complete resolution of these relationships and patterns of potential genetic exchange.

#### 3.2.7 Upland fuscus clade

A portion of the *fuscus* species complex comprising *fuscus* A, B, E, and *planiceps* is centered on the Blue Ridge Escarpment, though *fuscus* A ranges into the Interior Plateau and *fuscus* B ranges across the eastern US from the Interior Plateau to the Atlantic Ocean and north to eastern Canada. In contrast, *fuscus* E and *planiceps* are narrowly endemic to a portion of the Blue Ridge in northwestern North Carolina and southwestern Virginia (Beamer & Lamb, 2020; Tilley et al., 2008). All four are reciprocally monophyletic in the concatenated phylogeny, similar to their positions in previous studies. Selection of *K* using minimum cross‐entropy did not form an elbow, but yielded *K* = 6 by the notch test. The clustering and admixture analyses estimate a single distinct source of genomic ancestry for *fuscus* A, E, and *planiceps* while recovering significant phylogeographic structure in *fuscus* B, for which sNMF estimates three major lineages. Of these, *fuscus* B1 occurs along the Appalachian Mountains north to Canada and into the Interior Plateau, *fuscus* B2 occurs on the Atlantic side of the Appalachians, while *fuscus* B3 is restricted to a tiny portion of the Blue Ridge Escarpment in northwestern North Carolina (Figures 13 and 14). These lineages exhibit significant admixture for great distances along their various contact zones.

The purported effects of hybridization on phylogenetic inference are seemingly apparent here, as well. An early‐diverging specimen of *planiceps* contains ~35% admixture from *fuscus* B and E. None of the other sampled *planiceps* are heavily admixed, nor are any of our *fuscus* E samples. The earliest diverging *fuscus* A sample also contains ~25% ancestry from a mixture of the other three candidate species, but none of the other *fuscus* A individuals are heavily admixed. Finally, the earliest diverging specimens of *fuscus* B contain 45%–55% ancestry from *fuscus* E. While the various phylogeographic sublineages of *fuscus* B exhibit extensive admixture across their contact zones, none of the other specimens have significant ancestry from any of the other candidate species in this group. Thus, the four candidate species as previously defined all appear to be genetically cohesive and only hybridize on the margins of their range in geographic contact with the other lineages, and a few specimens have “streaks.” A notable exception to this is the previous finding that some populations of *fuscus* E possess mitochondrial haplotypes from *auriculatus* B/C (Beamer & Lamb, 2020; Pyron et al., 2020).

#### 3.2.8 Lowland fuscus clade

The second clade of *fuscus*‐group species (*auriculatus* A, B, and C; *fuscus* C and D) are distributed primarily in the Piedmont and Atlantic Coastal Plain south and east of the Blue Ridge Mountains. Within these five candidate species as previously defined, all are reciprocally monophyletic in the concatenated phylogeny, with *auriculatus* A forming the sister lineage to the remaining groups on a relatively long branch (Figures 3, 15, 16). Selection of *K* using minimum cross‐entropy did not form an elbow, but yielded *K* = 6 by the notch test. As noted above, *auriculatus* B & C are collapsed into *auriculatus* B/C by the clustering and admixture analyses. Finally, *fuscus* C contains three phylogeographic lineages; one in the northern Blue Ridge foothills of western North Carolina and eastern Tennessee (C2), one primarily in the southern Blue Ridge foothills of western North Carolina and the Piedmont and Coastal Plain of South Carolina (C3), and one primarily in the Piedmont and Coastal Plain of northern North Carolina and southern Virginia (C1).

These phylogeographic lineages exhibit significant admixture across large distances around their contact zones. They are not monophyletic in the phylogeny and appear to show the strong impact of hybridization on topological inference, wherein more heavily admixed individuals occupy earlier diverging positions along ladder‐like grades. Accordingly, the earliest diverging specimen of *fuscus* C contains ~40% ancestry from *auriculatus* B/C, while another early‐diverging sample has ~30%. Significant admixture is also observed between *fuscus* C & D and between *auriculatus* B/C and both *fuscus* C and D. Finally, one specimen of *auriculatus* A is estimated to have ~14% ancestry from *fuscus* C. While this does not meet our 20% threshold for significance, it is remarkable in potentially corroborating a previous finding of a sister‐group relationship between *auriculatus* A and *fuscus* C in a previous phylogenetic network analysis (Pyron et al., 2020), and mirrored by results from the formalin‐fixed historical specimens (see below), which are included on this plot (Figure 15) for visualization purposes.

#### 3.2.9 Ouachita clade

As in recent concatenated, species‐tree, and network analyses, *brimleyorum*, *valentinei*, and *valentinei* B form a monophyletic group (Pyron et al., 2020), unlike recent mitochondrial analyses in which *valentinei* +valentinei B and *brimleyorum* are the successive outgroups to the *conanti* species group (Beamer & Lamb, 2020; Pyron et al., 2020). Here, as in previous concatenated analyses (Pyron et al., 2020), the group is the sister lineage of the *conanti* +ocoee groups and their associated candidate species. In contrast, previous species‐tree and network analyses estimated it as the sister lineage to the *ocoee* group alone (Pyron et al., 2020). While it is possible that ILS explains this variation completely, the dramatically differing mitochondrial and nuclear concatenated, species‐tree, and network topologies suggest the influence of deep‐time reticulation, which will require additional genomic sampling and methodological attention to unravel. None of the species exhibit significant admixture (Figure 17) with *K* = 3 in the sNMF analyses, though our sample size for these candidate species is small (*n* = 4–5). Additional sampling of populations and individuals is desirable to evaluate possible gene flow between *valentinei* and *valentinei* B and possibly between either and *brimleyorum*.

#### 3.2.10 *ocoee* clade

The *ocoee* species group estimated here includes *apalachicolae*, *monticola* A/C & B, and *ocoee* D, E, and F/G/H, as in most previous mitochondrial (excluding *ocoee* D) and nuclear concatenated, species tree, and network analyses (Beamer & Lamb, 2020; Kozak et al., 2005; Pyron et al., 2020). Each candidate species is reciprocally monophyletic as previously defined, even with the substantially increased geographic sampling of individuals. In contrast, this expanded sampling reveals complex patterns of phylogeographic lineage divergence and admixture both across lineages within candidate species and between candidate species (Figures 18 and 19). Selection of *K* using minimum cross‐entropy did not form an elbow, but yielded *K* = 9 by the notch test. Furthermore, comparisons of these patterns to the topology further suggest the impact of hybridization on ladder‐like grades in the concatenated estimate.

The admixture analyses in sNMF estimate two phylogeographic lineages *ocoee* D1 & D2, the first in the foothills of the Blue Ridge mountains and the second in the Piedmont of western Georgia and extreme eastern Alabama. The two lineages exhibit extensive admixture across their contact zone in northeastern Georgia. The earliest diverging specimen of *ocoee* D1 actually represents Kozak et al. (2005)’s “*ocoee* C” and contains ~10%–20% ancestry from both *ocoee* E1 and F/G/H, despite being distantly related in this and most other phylogenies. This mitochondrial lineage (*ocoee* “C”) was not sampled in our previous work (Pyron et al., 2020), and we did not treat it as distinct here due to its limited sampling and close relationship with *ocoee* D in preliminary analyses. Another specimen of *ocoee* D1 also contains ~20% ancestry from *ocoee* E2, a phylogeographic lineage of *ocoee* E (see below), and several have streaks.

The previously defined candidate species *monticola* A/C & B are both reciprocally monophyletic sister lineages here, with a few admixed specimens at a narrow contact zone in northern Georgia at the transition from the Piedmont to the Blue Ridge mountains. Several *monticola* B specimens have streaks of ~20% non‐*monticola* ancestry. Whether this represents admixture from the other *ocoee* lineages, unaccounted for introgression from a non‐*ocoee* species, noise in the SNP data, or uncertainty from the admixture algorithm remains unclear.

The clade comprising *apalachicolae* and *ocoee* E & F/G/H (and interacting genetically with *ocoee* D) represents a difficult proposition for delimiting candidate species. First, *ocoee* F/G/H is the monophyletic sister lineage to the remaining candidate species, and none of the sampled specimens have significant ancestry from any other single lineage. As with the *monticola* B and *ocoee* D specimens described above, one *ocoee* F/G/H has a ~40% “streak” of non‐F/G/H ancestry. The geographically expanded *apalachicolae* (Beamer & Lamb, 2008) consists of two disjunct phylogeographic lineages, *apalachicolae* A1 comprising the originally described Coastal Plains populations in Alabama, Florida, and Georgia (see Means & Karlin, 1989), and *apalachicolae* A2 in the Blue Ridge foothills of north‐central Georgia. The mountain form (*apalachicolae* A2) is not monophyletic and exhibits extensive admixture with *ocoee* E2 (see below) which it contacts parapatrically. The southern form (*apalachicolae* A1) is monophyletic and nested within *apalachicolae* A2, and does not exhibit significant (i.e., >20%) admixture with any other lineage. A possible exception is the two earliest diverging specimens which are estimated to have ~10% ancestry from *apalachicolae* A2.

Finally, *ocoee* E contains two phylogeographic lineages, *ocoee* E1 & E2, in the southern Nantahala mountains of the Blue Ridge. The first, *ocoee* E1, is the more northerly and contacts *ocoee* F/G/H parapatrically, while *ocoee* E2 occurs near the Georgia/North Carolina border, parapatrically contacting *ocoee* E1 to the north, *apalachicolae* A2 to the south, and *ocoee* D1 to the east. Additional sampling is needed to clarify which lineages occur to the west of *ocoee* E2. Both *ocoee* E1 & E2 exhibit extensive admixture with each other across their contact zone. While several early‐diverging specimens of *ocoee* E1 exhibit estimated individual ancestry from *ocoee* F/G/H and *apalachicolae* A2, they do not meet our 20% threshold for further interpretation. In contrast, one specimen of *ocoee* E2 has ~20% ancestry from *apalachicolae* A2, and one specimen of *ocoee* E1 contains ~30% ancestry from *ocoee* D2. Taken at face value, these patterns suggest extensive genetic contact between these lineages over large distances.

#### 3.2.11 Balsam clade

The enigmatic clade of *ocoee* A & B is estimated here as the sister lineage of the *conanti* species group (Figure 3), as in previous species‐tree and some network analyses (TreeMix) based on a smaller AHE dataset (Pyron et al., 2020). This is in contrast to previous concatenated and other network (SNAq) analyses that estimate it as the sister lineage of the *ocoee* group, and mitochondrial estimates placing *ocoee* A–D as the sister lineage to all *conanti*, *fuscus*, and *ocoee*‐group species (Beamer & Lamb, 2020; Kozak et al., 2005; Pyron et al., 2020). The mitochondrial versus nuclear placements and the result of the previous TreeMix analysis strongly support a scenario of ghost admixture (Racimo et al., 2015; Zhang et al., 2019) from an early‐diverging, likely extinct lineage of *Desmognathus* (Pyron et al., 2020). The two candidate species are reciprocally monophyletic (*K* = 2) without any evidence of significant admixture (Figure 20). However, the *ocoee* B lineage is also implicated in significant allele sharing with some *conanti*‐group species (see results below). While we do not directly compare those candidate species here to estimate individual ancestry coefficients, the Dsuite results suggest an additional facet of complex history for this group to be examined in the future.

#### 3.2.12 *conanti* clade

Finally, the *conanti* species group (*conanti* A–F, ‘beta,’ ‘gamma,’ and *santeetlah*; possibly allied with *carolinensis*) represents perhaps the most complex and challenging set of candidate species and phylogeographic lineages. This group was first addressed in detail, in part, by Tilley et al. (2013), who referred to some of the lineages as “innominate forms” possibly representing “failed species.” Here, we estimate both geographic and genealogical coherence of each candidate species and associated phylogeographic lineages, bolstering previous conclusions that at least some of them may, in fact, represent “good” species (Pyron et al., 2020), albeit with complex patterns of ancestral or recent contact and hybridization. Selection of *K* using minimum cross‐entropy did not form an elbow, but yielded *K* = 10 by the notch test.

First, *conanti* A is monophyletic and contains two phylogeographic lineages, *conanti* A1 in the Blue Ridge foothills of northwestern South Carolina and western North Carolina, and *conanti* A2 in the Piedmont and Coastal Plain of western South Carolina and eastern Georgia. The two lineages exhibit extensive admixture across their contact zone in northwestern South Carolina. None of the sampled specimens have significant (i.e., >20%) individual ancestry from any other lineage, but the two earliest diverging specimens have ~10% from *santeetlah* and *conanti* F, respectively. A series of *conanti* A2 specimens also exhibit “streaks” of mixed ancestry along with admixture from *conanti* A1.

Similarly, the sampled specimens assigned to *santeetlah* form a monophyletic group, with most of those from the core range of the species centered on the Great Smoky Mountains exhibiting exclusive *santeetlah* ancestry. In contrast, the four earliest diverging specimens exhibit significant admixture with ‘beta,’ *carolinensis*, *conanti* F, and *conanti* A at the contact zones between those lineages and *santeetlah*. Accordingly, another deeply nested specimen exhibits significant individual ancestry from ‘gamma’ at the contact zone between the two lineages. The sampled specimens of the geographically distinct candidate species *conanti* E found exclusively in lowlands west of the Mississippi River are monophyletic and do not exhibit significant admixture from any other lineage, though the two individuals close to the Mississippi exhibit “streaks” comprising ~15%–25% of their total ancestry.

The southwestern Blue Ridge endemic ‘gamma’ and the widespread *conanti* B/C/D form reciprocally monophyletic sister candidate species. As previously defined based on more limited sampling (Beamer & Lamb, 2020; Pyron et al., 2020), *conanti* B/C/D contains two phylogeographic lineages corresponding roughly to *conanti* B/D and *conanti* C (Kozak et al., 2005). These lineages exhibit substantial admixture across a wide contact zone from southeastern Louisiana to eastern Tennessee; additional sampling in central Alabama is desirable to clarify patterns in the central part of the hybrid zone. The two earliest diverging specimens of *conanti* B/C/D exhibit significant admixture from ‘gamma’ and *santeetlah*, but no other lineage is represented significantly in the individual ancestry of the other sampled specimens. Similarly, several specimens of ‘gamma’ exhibit significant admixture from *conanti* B/D and *conanti* C, while one specimen possesses ~10% of individual ancestry from ‘beta,’ a result estimated in previous analyses of this group (Tilley et al., 2013). None of the sampled specimens of ‘gamma’ contain significant individual ancestry from any other lineage. We note that *conanti* B/C/D was previously estimated to have arisen via hybridization between ‘gamma’ and *ocoee* F/G/H (Pyron et al., 2020), but this was not estimated in our Dsuite admixture analyses (see below).

Finally, the sampled individuals of ‘beta’ and the primary monophyletic group of sampled *conanti* F specimens are genealogically exclusive and do not contain any significant or notable individual ancestry from any other lineage. In contrast, four specimens assigned to *conanti* F *a priori* (three of which form a clade) are more closely related to *carolinensis* in the concatenated phylogeny. These individuals have significant genomic ancestry from *conanti* F, *carolinensis*, ‘beta,’ and *conanti* A, and notable amounts (~10%) from *conanti* E. Similarly, the earliest diverging specimen of *carolinensis* contains notable admixture (~10%) from ‘beta’ and *conanti* F, respectively, while several other *carolinensis* individuals also possess ~10%–20% individual ancestry from ‘beta.’ These admixed individuals occur around the geographic contact zones between these lineages (except the distant *conanti* E), suggesting complex hybridization or other genomic admixture dynamics in southwestern North Carolina. In contrast, at least one specimen with ~100% *conanti* F ancestry occurs sympatrically with specimens exhibiting ~100% *conanti* A2 ancestry, suggesting that these lineages can nonetheless maintain genetic distinctiveness in close geographic proximity.

### 3.3 Admixture and reticulation

The results from Dsuite (Figure 23) corroborate several previously estimated or hypothesized instances of gene flow in *Desmognathus*, and present additional insight for future targeted analyses. The matrix of *f*_b_ values can be read to indicate the proportion of alleles shared between a donor species in the columns and the recipient branch in the rows in excess of that predicted by the MSC model (Malinsky et al., 2021). The recipient branches can be either internal or terminal, and the matrix is, therefore, partially symmetric, as the terminal branches are present on both axes. Directionality can be difficult to determine without additional testing (Pease & Hahn, 2015; Svardal et al., 2020); we remain agnostic on this question in most instances. Additionally, a single instance of gene flow can produce a correlated signal of non‐zero *f*_b_ values across multiple lineages related to the donor, resulting in a horizontal line of significant inference within a row, providing limited interpretability. Finally, we only present *f*_b_ values above 20% for comparison with the threshold we set for individual ancestry coefficients. We find 11 recipient branches involved in eight apparently distinct sets of hybridization events resulting in excess allele sharing from up to 16 terminal candidate species, although the actual number of donors is likely much smaller, based on the artifacts described above.

First is the complex interplay within and between candidate species from *ocoee* and *conanti* lineages, part of which was captured above in our admixture analyses. Within the *conanti* group, most of the admixture patterns described previously are captured in the Dsuite analyses; admixture between ‘beta’ and *conanti* F (64%) being supported most strongly, but with *conanti* B/C/D, ‘gamma,’ *santeetlah*, and *conanti* A also being implicated.

A second pattern that has not been estimated previously is gene flow to the *conanti* species group from the Balsam clade (*ocoee* A & B), most strongly from *ocoee* B. Given the partial symmetricity of the matrix, the reverse scenario is also possible, with *ocoee* B receiving alleles from one or more *conanti*‐group lineages. This is likely related to the ghost admixture scenario estimated in a previous network analysis in TreeMix (Pyron et al., 2020).

Third, several species in the *ocoee* clade (*apalachicolae* and *ocoee* D, E, and F/G/H) are all implicated in contributing alleles to species in the Balsam and *conanti* clades. As this result is not significantly symmetric, the directionality may be more meaningful. This partially corroborates previous network analyses in SNAq that estimated *conanti* B/C/D arising as the result of a hybrid speciation event between *ocoee* F/G/H and ‘gamma’ (Pyron et al., 2020).

A fourth and related pattern of particular importance involves *fuscus* C as a recipient branch from *conanti* F and ‘beta.’ Many populations of *fuscus* C share mitochondrial haplotypes with *carolinensis* (Beamer & Lamb, 2020; Kozak et al., 2005; Pyron et al., 2020), here estimated as the sister lineage of *conanti* F, but the alleles shared between them are not significant in the Dsuite analysis. However, our previous TreeMix analysis of a smaller AHE dataset estimated a three‐way reticulation among *carolinensis*, *conanti* F, and *fuscus* C, which is, thus, partially corroborated here. We note again, however, that not all significant lineages are necessarily donors, based on the potential artifacts described above from branch correlations. In the population‐level admixture analyses, we considered the *fuscus*, *conanti*, and *ocoee* species groups separately; future analyses will need to analyze them jointly to unravel these patterns.

A fifth more isolated instance of allele sharing is estimated between *fuscus* E and *planiceps*, a finding which also occurs in the admixture analyses (see above; Figure 14; Tilley et al., 2008). Sixth, *orestes* B & AC share a 26% excess of alleles, a pattern clearly established in the admixture analyses (Figure 11) and previous analyses (Mead et al., 2001). Seventh, *marmoratus* E/H and *quadramaculatus* D are estimated to share excess alleles, which is reflected at low frequencies in our admixture analyses (Figure 8) and potentially related to the sharing of mitochondrial haplotypes between *marmoratus* E/H and *quadramaculatus* E (Kozak et al., 2005). Finally, the eighth major event is significant gene flow between *quadramaculatus* E & G (44% excess alleles), which is strongly corroborated in the admixture analyses (Figure 9).

### 3.4 Formalin‐fixed sequencing

As noted above, extraction and library preparation for the two fluid‐preserved specimens was modestly successful but yielded sufficient coverage for only 55% of the loci (129/233) and just 5% of the total alignment (27,763/563,656 bp). Pruning this reduced alignment to the relevant clades (*auriculatus* A, B/C; *fuscus* C and D) for clustering analysis yielded 3126 variable sites. However, the two specimens (USNM 468094–5) only overlapped at a few sites, because successful reads for each specimen generally mapped to different loci or different parts of the same locus. We, therefore, divided the dataset into fully sampled SNP matrices for each specimen, yielding 1176 SNPs from 56 loci for USNM 468094 and 1605 from 79 for USNM 468095. The phylogenetic and clustering analyses both strongly estimated membership of these specimens in *auriculatus* A (Pyron et al., 2022). Both analyses also estimated the same general clusters and relationships as the primary analysis of the Lowland *fuscus* clade, with *auriculatus* B/C supported as a single candidate species along with *fuscus* D, and three admixed phylogeographic lineages in *fuscus* C (Figure 15; see full results in Dryad repository https://doi.org/10.5061/dryad.f4qrfj6x8).

Intriguingly, the sNMF admixture analysis suggested a non‐zero percentage of *fuscus* C ancestry in USNM 468094 (16%) and 468095 (25%), the existence of which was hypothesized in our previous study (Pyron et al., 2020). However, we also noted long terminal branches for these specimens in the concatenated ML phylogenetic estimate (see SI) and a large number of unique, apparently homozygous SNP calls in these specimens (Pyron et al., 2022), potentially driven by DNA degradation from fixatives or preservatives (O’Connell et al., 2021). Thus, the branch lengths and “*fuscus* C” ancestry may be noise arising from template or sequencing errors misleading the sNMF algorithm. Future analyses can test the hypothesis of DNA damage (or other processes such as sequencing or assembly error) by attempting to sequence more loci to greater depth while accounting for DNA degradation (Ginolhac et al., 2011). We did not attempt this here due to the limited extent of capture success for these specimens.

## 4 DISCUSSION

We analyzed a dataset containing 896 samples from 732 sites, including all 49 mito‐nuclear candidate species of *Desmognathus* sampled for up to 233 Anchored Hybrid Enrichment (AHE) loci. This study had three primary aims: (i) to objectively evaluate the distinctiveness of the 49 previously delimited mito‐nuclear candidate species, (ii) to estimate the presence of phylogeographic lineages therein and the prevalence of admixture both within and between candidate species, and (iii) to estimate the possible impact of both deep‐time reticulation and recent gene flow on topological estimates within and among clades. We estimate 47 candidate species supported by monophyly in the concatenated ML topology of the AHE dataset, all of which coincide with estimates of distinct genomic ancestry and partial to complete genealogical exclusivity in the admixture analyses. However, many of the 47 candidate species contain significant geographic genetic structure, with up to 30 phylogeographic lineages within 13 of those 47 taxa, and therefore as many as 64 geographically and genetically distinct population segments within *Desmognathus* (Table 1). Additional data are needed to make final determinations regarding taxonomic status for these lineages and provide more detailed estimates of their history of divergence and reticulation. Nonetheless, these results represent a significant step forward for this historically problematic genus and a robust foundation for future analyses.

Our estimates of candidate species, most or all of which likely merit taxonomic recognition as distinct species, appear to be stable. In contrast, many of these lineages contain extensive genetic structure across geographic space apparently resulting from complex phylogeographic histories, future study of which will likely yield rich insights into speciation processes in the group. As noted above, we refer to distinct population clusters estimated by the admixture analyses, but which intergrade extensively across their contact zones and do not form reciprocally monophyletic groups in the phylogeny, as phylogeographic lineages.

Crucially, a series of recent empirical studies have highlighted a potentially artifactual process by which species delimitation methods that do not account for gene flow may erroneously inflate estimates by identifying hybrid populations as distinct taxa (Chan et al., 2017, 2020, 2022; Dolinay et al., 2021). Admixed populations resulting from spatiotemporally proximate hybridization events may consequently produce individuals with similar allele frequencies, distinct from either parental population, that are therefore grouped by clustering and phylogenetic analyses. These populations may even possess distinct mitochondrial haplotypes, not because they are truly distinct evolutionary lineages, but which were instead captured asymmetrically from other lineages during introgression events (Mastrantonio et al., 2016).

We were initially concerned that detailed analyses might reveal such an artifactual origin of many previously delimited candidate species. However, the effect seems limited to two primary instances. The first is *marmoratus* “G,” which occupies an intermediate topological position between *quadramaculatus* G and *marmoratus* C + *quadramaculatus* C, while possessing a genome apparently composed of contributions from all Pisgah lineages except *quadramaculatus* E1, a small plurality of which is from *marmoratus* C. Similarly, populations of *marmoratus* “G” have mitochondrial haplotypes closely related to those of *quadramaculatus* G, suggesting that they were captured during a past introgression event involving *quadramaculatus* G and *marmoratus* C, at a minimum. Consequently, *marmoratus* “G” may not, in fact, represent a distinct candidate species. Second, a cluster of four individuals previously assigned to *conanti* F form a clade occupying a distinct topological position as the sister lineage to *carolinensis*, rather than nesting within the other sampled specimens of *conanti* F. Accordingly, the admixture analyses reveal them to have hybrid ancestry consisting primarily of *conanti* F and *carolinensis*, but also potentially *conanti* A, E, and ‘beta.’

While admixture between genealogically distinct parental populations does not appear to significantly confound delimitation of candidate species in our analyses, it does seem to exert a strong influence on phylogenetic topologies (Degnan, 2018). An extension of the artifacts first reported by Chan et al. (2020, 2022) and demonstrated by Dolinay et al. (2021) suggests that hybrid individuals will form ladder‐like grades of intermediate topological position between “pure” parental populations in rough proportion to their degree of ancestry from each parent. This effect is corroborated strongly in our analyses, where the topological imbalance of candidate species is significantly related to their degree of admixture. In nearly all clades, highly admixed individuals occupy early‐diverging positions in their primary candidate species as they are seemingly pulled toward their secondary candidate species in the tree. This is particularly notable in *orestes*, *conanti*, *carolinensis*, *fuscus*, and *ocoee*. This is essentially the originating process at the population level which ultimately produces the species‐delimitation artifacts originally described by Chan et al. (2020, 2022); sampling more highly admixed individuals from the clades mentioned would likely end up producing entire false clades in estimated trees.

Finally, these same processes appear to be influencing topological estimation of deeper nodes, with ancestral relationships confounded by deep‐time reticulation as described by numerous recent authors (Burbrink & Gehara, 2018; Knowles et al., 2018; MacGuigan & Near, 2019). Our previous network‐based analyses resolved several instances of gene‐tree congruence between mitochondrial and nuclear concatenated and species‐tree analyses that apparently resulted from ancestral hybridization (Pyron et al., 2020). In addition to ILS, discordance is likely due to the widespread and pervasive impact of introgression across numerous lineages and timescales, evident in both network analyses of unlinked nuclear loci and in mitochondrial genome capture in extant populations (see similar examples in Çoraman et al., 2019; Li et al., 2019; Weisrock et al., 2005). Mitochondrial capture between the stem lineages of the Nantahala and Pisgah clades, ghost admixture of the *ocoee* A, B, C, and D lineages, and the varying placement of *carolinensis* are key examples. The complex dynamics of the latter two are partially illuminated by our tree‐based admixture analyses in Dsuite, although a comprehensive model for all of them is still lacking.

Essentially, our results and those of recent authors such as Chan et al. (2022) and Dolinay et al. (2021) reveal the need for an analytical and computational framework that can simultaneously estimate complex phylogenetic networks and delimit candidate species with gene flow. Few if any such methods exist, especially in a computationally tractable form for a dataset of this size, containing hybridizations across multiple distant phylogenetic scales. We have attempted to integrate multiple lines of evidence to provide a rough sketch of these dynamics in *Desmognathus*, although more data will be needed to provide more definitive explanations for many of the complex patterns described above. Additional questions of interest at the nexus of phylogeny and phylogeography (Edwards et al., 2016) include distinguishing between primary versus secondary contact (Feder et al., 2013), ancient versus recent admixture (McTavish & Hillis, 2014), and ILS versus introgression (Schaefer et al., 2021; Wang et al., 2018; Zhou et al., 2017). All of these will be crucial in future analyses to better understand *Desmognathus* relationships, speciation processes, and phylogeographic histories.

### 4.1 Challenges for understanding species limits

Species delimitation is a challenge that cannot be easily settled by computational algorithms alone (Carstens et al., 2013; Padial et al., 2010; Sukumaran & Knowles, 2017). The interplay of stochastic coalescent variation (Knowles & Carstens, 2007), introgression (Martin et al., 2013), and spatial and ecological barriers to gene flow (Burbrink et al., 2021) can yield strikingly complex scenarios in the “gray zone” of speciation (Matute & Cooper, 2021; de Queiroz, 2007). Accordingly, a range of these scenarios is observed here in *Desmognathus*. Many if not most candidate species are topologically, genetically, and geographically cohesive. As such, they are spatiotemporally distinct ontological individuals in the evolutionary sense (Ghiselin, 1974; Hull, 1976), and most will likely be recognized as distinct species in future taxonomic revisions. In contrast, we highlight three major groups of candidate species described above presenting additional challenges for interpretation.

The first is the Pisgah clade of *marmoratus* and *quadramaculatus* sublineages. Our results suggest complex dynamics potentially indicating a “network radiation” (Kozak et al., 2021) consisting of speciation by hybridization (Mavárez et al., 2006) resulting from genomic processes (Abbott et al., 2013). Regardless, the six lineages estimated here by clustering admixture analyses are reciprocally monophyletic, morphologically diagnosable in some cases (*marmoratus* vs. *quadramaculatus*), and appear to have clear genetic and geographic boundaries as candidate species, albeit permeable ones (Harrison & Larson, 2014). Therefore, we continue to recognize them as candidate species (with *quadramaculatus* E containing two phylogeographic lineages), but with *marmoratus* G having perhaps the strongest evidence for being of hybrid origin, and therefore potentially being considered conspecific with *marmoratus* C. However, the difficulty of treating multiple morphologically similar but distantly related candidate species that hybridize in parapatry without any apparent geographic, ecological, or microhabitat partitioning (e.g., *quadramaculatus* C, E, and G) is a thorny conceptual challenge.

A similar situation arises in the *ocoee* clade, with respect to *apalachicolae* and *ocoee* D, E, and F/G/H. These candidate species and their constituent phylogeographic lineages each exhibit signatures of geographically distinct genomic ancestry, while simultaneously exhibiting admixture across broad parapatric contact zones in the southern Blue Ridge mountains. While some peripheral, Piedmont, and Coastal Plains populations exhibit more distinctive phenotypes (Means & Karlin, 1989; Valentine, 1961), the Blue Ridge populations are essentially indistinguishable phenotypically. Given the spatial and genetic complexity of historical evolutionary relationships in this clade, additional genetic and population sampling will be required to establish robust species limits in the group. We continue to treat the candidate species as distinct, with phylogeographic lineages in *ocoee* D and E. Considering the extensive admixture of *apalachicolae* A2 with *ocoee* E2 and the exceptional geographic separation of *apalachicolae* A1, we consider *apalachicolae* A1 alone to represent the species *Desmognathus apalachicolae* (Means & Karlin, 1989) as originally described in the Coastal Plain of Georgia, Alabama, and Florida. This reverses the earlier conclusions of Beamer and Lamb (2008) based solely on mitochondrial data. Thus, *apalachicolae* A2 likely represents either an additional phylogeographic lineage of *ocoee* E (which would then be paraphyletic, albeit in a topology we suspect to exhibit significant artifacts arising from gene flow), or possibly an additional distinct candidate species endemic to the foothills of the Blue Ridge in Georgia.

A third major instance of these patterns occurs with the *conanti* species group (Figures 21 and 22), including *santeetlah* as well as *carolinensis*, estimated as a member of this clade here for the first time. Several of the candidate species are mostly geographically and genetically distinct and cohesive, including *conanti* E west of the Mississippi River, *conanti* B/C/D in the Interior Plateau and Gulf‐draining Piedmont and Coastal Plain, and *conanti* A in the Savannah River drainage of the Atlantic Piedmont and Coastal Plain. In contrast, the montane candidate species of the southern Blue Ridge (*santeetlah*, ‘gamma,’ ‘beta,’ *conanti* F, and *carolinensis*) all exhibit extensive admixture at their contact zones in parapatry, including a region in southwestern North Carolina characterized by 4‐ or 5‐way hybrids in our admixture analyses. Interaction between this group and the *ocoee* A & B and F/G/H lineages and *fuscus* C is also indicated by our Dsuite analyses and previous network estimates (Pyron et al., 2020) and mitochondrial and allozyme data (Tilley et al., 2013).

In contrast to the *ocoee* group described above but more similar to the Pisgah clade, the *conanti*‐group candidate species in the southern Blue Ridge exhibit several distinct phenotypes, including a smaller “mountain dusky” morphology with a round tail (‘beta,’ *carolinensis*, and some *conanti* A and F populations), a larger “dusky” morphology with a laterally compressed tail (‘gamma,’ *conanti* B/C/D), and the distinctively colored *santeetlah* (Petranka, 2010; Pope, 1924; Tilley, 1981; Tilley et al., 2013). Determining the precise evolutionary history of these populations and their interactions will require additional genomic and population‐level sampling. Some empirical and theoretical research has even suggested that, through a series of complex demographic, genomic, and population‐genetic processes, such hybrid populations as those observed here may actually serve to filter gene flow between species (Martinsen et al., 2001). This may lead to stable hybrid zones that promote adaptive introgression and prevent lineage collapse (Barth et al., 2020); studying these dynamics (selection in particular) may be revealing for *Desmognathus* as in other salamanders (Alexandrino et al., 2005; Johnson et al., 2015).

Precise definitions of species limits and descriptions of potentially new species remains a challenge for future studies, although most candidate species demonstrate substantial geographic, genetic, and morphological distinctiveness. As noted above, the Dsuite analyses, previous network analyses (Pyron et al., 2020), and the varying placements of *ocoee* A & B and *carolinensis* across studies suggest cross‐clade interactions among the *conanti*, *fuscus*, and *ocoee* groups that were not captured by the design of our clade‐specific admixture analyses. There are also additional apparent instances of mitochondrial genome capture (e.g., between *auriculatus* C and *fuscus* E, between *fuscus* B and D, and between the stem lineages of the Nantahala and Pisgah clades) that have yet to be estimated in any network or admixture analyses. There are numerous other putative instances of hybridization between distantly related but sympatric or parapatric species pairs (see reviews in Beamer & Lamb, 2020; Tilley, 2016). However, most of these typically occur at low frequency and are generally based on allozyme analyses which are susceptible to electromorphic homoplasy and consequently may not represent real introgression in some cases (Henriques et al., 2016).

### 4.2 Historical specimens of extirpated populations

In the sNMF admixture analyses, both fluid‐preserved specimens were estimated to have a substantial amount (~20%–40%) of hybrid ancestry from some *fuscus* C sublineages, along with one of the modern samples (Figure 15). Our previous phylogenetic network analysis (Pyron et al., 2020) using PhyloNetworks (Solís‐Lemus & Ané, 2016) actually estimated a sister relationship between *auriculatus* A and *fuscus* C, with the ancestor of the pair receiving 32% of its ancestry from *carolinensis*. We noted that this relationship was not reflected in our other phylogenetic analyses at the time, nor is it estimated here among our recent specimens in terms of topology, clustering, or admixture, or by any mito‐nuclear discordance.

However, we also noted that the extinction of numerous *auriculatus* A populations (such as the former *fuscus* subspecies “*carri*”) may have limited our ability to recover this signal (Pyron et al., 2022). Therefore, the signal of *fuscus* C ancestry in extirpated peninsular populations of *auriculatus* A may indeed be a real pattern reflecting historical evolutionary relationships and hybridization. Alternatively, it may reflect the known high error rate of sequencing for formalin‐fixed specimens (Hykin et al., 2015; Oh et al., 2015), or poor signal from our small dataset. One strategy may be to combine multiple extractions to increase input DNA into capture reactions and sequence available fragments at greater depth. Hopefully, future improvements in extraction and sequencing technologies will increase efficiency and reduce error rate for fluid‐preserved specimens and shed additional light on the genomics of these enigmatic extinctions.

## 5 CONCLUSIONS

Our aim in this study was to (i) evaluate the distinctiveness of the 49 previously defined mito‐nuclear candidate species of *Desmognathus* or the presence of any new such groups, (ii) examine these putative taxa for additional phylogeographic lineages and the existence and extent of hybrid zones, and (iii) assess the impact of introgression on the reconstruction of bifurcating phylogenetic topologies. We find that previous estimates have converged on a roughly stable estimate of species‐level diversity in the genus and corroborate the existence of 47 candidate species. Many of these candidate species exhibit extensive admixture with each other along their geographic margins, and in many cases with non‐sister or even distantly related clades. Similarly, many candidate species contain significant geographic genetic structuring, with multiple phylogeographic lineages exhibiting broad hybrid zones. This extensive gene flow across species boundaries even at great phylogenetic distance apparently exerts a strong influence on topological reconstructions, both the placement of terminal specimens and entire clades. Concatenated, species‐tree, and network analyses have yet to conclusively resolve the placement and relationships of groups, such as *abditus*, *carolinensis*, *ocoee* A & B, and *brimleyorum* + *valentinei*. Similarly, a satisfactory model of species limits in the *conanti*, *ocoee*, and Pisgah clades in particular will require additional genomic and geographic sampling, along with more computationally sophisticated and biologically realistic models. Our results here provide a comprehensive if basic evaluation of the landscape of genetic diversity in *Desmognathus* and should support these future studies in targeting further comparisons.

## CONFLICT OF INTEREST

The authors declare that they have no conflict of interest.

## AUTHOR CONTRIBUTIONS

**R. Alexander Pyron:** Conceptualization (equal); Data curation (equal); Formal analysis (equal); Funding acquisition (equal); Investigation (equal); Methodology (equal); Project administration (equal). **Kyle A. O’Connell:** Conceptualization (equal); Data curation (equal); Formal analysis (equal); Investigation (equal); Methodology (equal); Project administration (equal). **Emily Moriarty Lemmon:** Data curation (equal). **Alan R. Lemmon:** Data curation (equal). **Dave A. Beamer:** Conceptualization (equal); Data curation (equal); Formal analysis (equal); Funding acquisition (equal); Investigation (equal); Methodology (equal); Project administration (equal).

## Data Availability

All alignments, code, and results described in this paper are available in the DataDryad repository https://doi.org/10.5061/dryad.f4qrfj6x8. This includes the AHE assemblies and SRA accessions, specimen data and localities, custom R scripts for analysis, and the phylogenetic, clustering, and admixture results. The raw reads for the AHE assemblies are available in SRA PRJNA776902, PRJNA778238, PRJNA778269, PRJNA778013, and PRJNA777585.
